# Entanglement-controlled vectorial meta-holography

**DOI:** 10.1038/s41377-025-01818-w

**Published:** 2025-03-25

**Authors:** Sheng Ye, Yue Han, Li-Zheng Liu, Weiping Wan, Ruiqi Wang, Mingna Xun, Qiang Li, Qihuang Gong, Jianwei Wang, Yan Li

**Affiliations:** 1 State Key Laboratory for Artificial Microstructure and Mesoscopic Physics, School of Physics, Peking University, Beijing, 100871 China; 2 Frontiers Science Center for Nano-optoelectronics & Collaborative Innovation Center of Quantum Matter, Peking University, Beijing, 100871 China; 3 Collaborative Innovation Center of Extreme Optics, Shanxi University, Taiyuan, 030006 Shanxi China; 4 Peking University Yangtze Delta Institute of Optoelectronics, Nantong, 226010 Jiangsu China; 5 Hefei National Laboratory, Hefei, 230088 China

**Keywords:** Quantum optics, Metamaterials, Imaging and sensing

## Abstract

Metasurfaces can precisely manipulate the amplitude, phase, and polarization of incident light through subwavelength structures, greatly advancing the quantum meta-holographic imaging. However, the current methods of using quantum holography only control either the amplitude or the phase on the imaging plane, so the resulted scalar holography without the polarization distribution has limited imaging channels. Here, the vectorial meta-holography using entangled signal-idler photon pairs is experimentally demonstrated to realize remotely controlled multi-channel quantum imaging. By simultaneous control of the amplitude ratio between two cross-polarization holographic images and their phase difference on the image plane, the polarization distribution accordingly changes with the incident polarization state. The accurate correspondence ensures the correct reconstruction of 32 incident polarization states with an average fidelity up to 94.78%. This enables entangled idler photons to remotely control the holographic images reconstructed by the entangled signal photons, where the signal-to-noise ratio is as high as 10.78 dB, even for maximally mixed quantum states. This vectorial meta-holography using entangled states has a larger polarization state information capacity and will facilitate miniaturized quantum imaging and efficient quantum state tomography.

## Abstract (web summary)

We experimentally demonstrate the entanglement-controlled vectorial meta-holography for precise quantum state reconstruction and high SNR image remote control via idler photon polarization manipulation.

## Graphical abstract

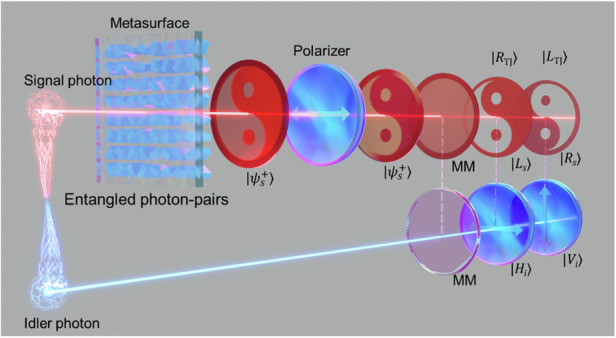

## Introduction

Metasurfaces have shown great potential in replacing multiple bulky optical devices with a two-dimensional planar structure of subwavelength thickness^1,2^, providing precise control over amplitude, phase, and polarization of electromagnetic waves^3–5^ to manipulate various degrees of freedom of photons, including path^6^, wavelength^7^, and orbital angular momentum (OAM)^8–10^. Nowadays, a wide range of metasurface-driven applications have been reported, such as dispersion engineering^11,12^, nano-optical sensing^13,14^, and holographic imaging^15–17^.

The meta-holography not only enables smaller pixel sizes, a wider field of view (FoV) and broader bandwidth, but also provides a unique strategy for other pivotal applications^18^. Harnessing the quantum correlation of entangled photons, quantum imaging can effectively improve the signal-to-noise ratio (SNR)^19–21^ and the spatial resolution^22,23^, which substantially determine the performance and quality of the desired imaging^24^. The observed evident enhancements in resisting classical noise (e.g. stray light and dark counts)^25–27^ come from the second-order degree of the optical coherence of quantum states instead of its first-order degree^28^. The imaging SNR enhancement is attributed to the coincidence of entangled photons, which effectively blocks the uncorrelated classical noise^29^. With a longer integration time, the results are closer to the theoretical expectations, although the noise also increases linearly during the integration process. Additionally, the quantum meta-holography can directly identify the optical polarization state^30–32^ by correlation measurements of entangled photons, without the need for the interference between the object and an additional reference beams in traditional holography. Furthermore, it gets rid of the polarizer usually required at the imaging terminal^33^. Altuzarra et al. multiplex 2 orthogonal polarization states for 2 imaging channels using entangled photons. In the classical domain, the number of imaging channels can be achieved as high as 36^3^. However, the application of quantum holography is currently limited by the inadequate imaging channels, the time consuming for image acquisition^34^ and challenges in quantitative analysis of experimental data^35^.

**Fig. 1:**
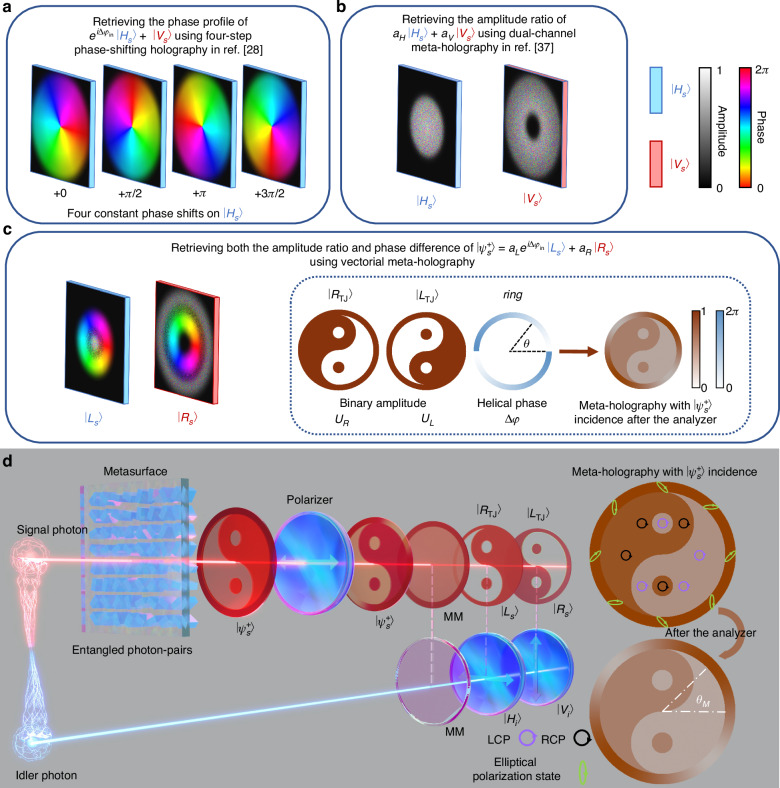
Schematic of entanglement-controlled vectorial meta-holography (ECVMH) **a** Schematic of method for retrieving phase of incident horizontally polarized states$\,\left|{H}_{s}\right\rangle$. A holographic image (a color helical palette) is reconstructed through a four-step process that retrieves the phase of incident single polarization state $\left(\left|{H}_{s}\right\rangle\right.$ and $\left.\left|{V}_{s}\right\rangle\right)$, with its near-unity amplitude. **b** Schematic of method for retrieving amplitude ratio of incident cross-polarized states $\left(\left|{H}_{s}\right\rangle\right.$ and $\left.\left|{V}_{s}\right\rangle\right)$. The average intensities of holographic images from dual polarization states corresponds to their amplitude ratio, but the phase distribution remains random, even within the overlapped region. **c** Schematic of vectorial holography for retrieving both phase difference and amplitude ratio of two incident cross-polarized states by spatial multiplex of two metaholograms for $\left|{L}_{s}\right\rangle$ and $\left|{R}_{s}\right\rangle$ into a single metasurface. The inset shows the principle of the vectorial meta-holography. The phase difference is retrieved by the outer ring with spatially variant intensity distribution after the analyzer; while the complementary images are independent by letting their phases to be random, so their intensity ratio can derive the amplitude ratio of the two incident cross-polarized states. **d** The ECVMH is realized by the vectorial meta-holography and the incident quantum entangled photon-pairs ${|\Phi }_{\psi }^{+}\rangle$. When the signal photon is in $|{\psi }_{s}^{+}\rangle$ state, the corresponding holographic image is shown in before and after the analyzer. The upper right inset shows the vectorial meta-holography when the signal photon collapses into arbitrary polarization state $|{\psi }_{s}^{+}\rangle$, and the lower right inset displays its intensity distribution after the horizontally oriented analyzer. When $|{\Phi }_{\psi }^{+}\rangle =|{\Phi }_{{LR}}^{+}\rangle$ and the idler photon in blue is triggered to a certain polarization state, the reconstructed meta-holography of signal photons after the analyzer in red accordingly changes, whose states are in MM, $\left|{L}_{s}\right\rangle$, and $\left|{R}_{s}\right\rangle$ states

Although the quantum holography is usually hard to retrieve the information of the incident states, it has been made significant methods as reported in recent two noteworthy works with high noise resistance. Defienne et al. enhance the spatial resolution of the holographic image using spatial-polarization hyper-entangled states, even in the presence of dynamic phase disorder and strong classical noise^28^. By utilizing a four-step phase-shifting holography^36^, the phase profile of signal photons can be reconstructed from near-unity amplitude images by manipulating the phase distributions of incident horizontally polarized idler photons, as shown in Fig. 1a. For eliminating the noise and improving contrast, Fan et al. realize remote switching of dual-channel meta-holography using polarization entangled states^37^. This method of orthogonal polarization multiplexed imaging channels can be used for retrieving the amplitude ratio of cross-polarized incident photons, as shown in Fig. 1b. However, the quantum holography captured by a camera demands substantial computational resources for data processing and storage to accurately retrieve the phase and the intensity. In the meantime, the methods of retrieving only phase or amplitude ratio of incident photons restricts the reconstruction of arbitrary polarization state, thereby limiting the polarization-encoded quantum channels and, consequently, the imaging channels^38^.

In this work, we demonstrate the entanglement-controlled vectorial meta-holography (ECVMH) as shown in Fig. 1c, d, for the first time, that dynamically controls polarization entangled imaging using a Pancharatnam-Berry (PB) phase-based dielectric metasurface with high-transmittance. Thanks to precise manipulation of the polarization distribution via controlling both the phase difference and the amplitude distribution on the imaging plane, the quantum-enhanced vectorial meta-holography can reconstruct arbitrary incident polarization-encoded information, even for maximally mixed (MM) states. For 32 distinct polarization states, the average fidelity is up to 94.78%. Using entangled photon-pairs, remote trigger of the idler photon to different polarization states activates the signal photon to generate SNR-enhanced quantum holography.

## Results

### Design of the entanglement-controlled vectorial meta-holography

An arbitrary incident polarization state $\left|{\psi }_{s}^{+}\right\rangle$ (e.g., an elliptical polarization state) can be written as the superposition of $|{L}_{s}\rangle$ and $|{R}_{s}\rangle$ states as$\,\left|{\psi }_{s}^{+}\right\rangle ={a}_{L}{e}^{i\Delta {\varphi }_{{\rm{in}}}}\left|{L}_{s}\right\rangle +{a}_{R}\left|{R}_{s}\right\rangle$, where $|{L}_{s}\rangle \,$ and $|{R}_{s}\rangle$ are the left circularly polarized (LCP) and the right circularly polarized (RCP) states, and ${a}_{L},{a}_{R},$ and$\,\Delta {\varphi }_{{\rm{in}}}$ denote the amplitudes of LCP, RCP components and their phase difference, respectively. As presented in Fig. 1c, they are retrieved by specially designed vectorial meta-holography for $|{L}_{s}\rangle$ and $|{R}_{s}\rangle$ as explained as follows.

In the inset, both meta-holography images contain two parts, the inner Tai Chi Symbol and an outer ring. The binary Tai Chi Symbol or so-called “*Yin*/*Yang*” Symbol consists of a circle divided into two complementary halves by a curved line. One half of the circle is dark in white, typically representing the “*yin*” side; the other is bright in red, for the “*yang*” side. When only $|{L}_{s}\rangle$ or $|{R}_{s}\rangle$ component is incident on the metasurface, it is converted into the complementary inner Tai Chi Symbol and the outer ring with RCP or LCP state, denoted as $\left|{R}_{{\rm{TJ}}}\right\rangle ={U}_{R}{e}^{i{\varphi }_{R}}\left|R\right\rangle \,$ or $\left|{L}_{{\rm{TJ}}}\right\rangle ={U}_{L}{e}^{i{\varphi }_{L}}\left|L\right\rangle$ (see Supplementary Information 2). The generated $\left|{R}_{{\rm{TJ}}}\right\rangle$ and $\left|{L}_{{\rm{TJ}}}\right\rangle$ are accompanied by an unconverted zero-order output retaining LCP or RCP state^39^. In the following, we use |*R*〉 and |*L*〉 to represent the polarization state on the imaging plane, while the incident states are denoted as $|{L}_{s}\rangle$ and $|{R}_{s}\rangle$.

In the inner region of the Tai Chi Symbol, two images with random phases for both $\left|{R}_{{\rm{TJ}}}\right\rangle \,$ and $\left|{L}_{{\rm{TJ}}}\right\rangle$ are generated and overlapped when an arbitrary state $\left|{\psi }_{{\rm{s}}}^{+}\right\rangle$ is incident. Analyzed with a linear analyzer oriented at an angle $\alpha$ (typically $\alpha =0$), their average intensities ${I}_{R}\,$ and ${I}_{L}$ of the bright area are proportional to the incident intensities ${a}_{L}^{2}$ and ${a}_{R}^{2}$, respectively. Because the cross-polarization conversion ratio (CPCR) for incident ${{|L}}_{s}\rangle$ and $|{R}_{s}\rangle \,$ state is identical and the bright area of both $\left|{R}_{{\rm{TJ}}}\right\rangle \,$ and $\left|{L}_{{\rm{TJ}}}\right\rangle$ images are equal, the amplitude ratio of the incident LCP and RCP states can be obtained by ${a}_{L}/{a}_{R}=\sqrt{{I}_{R}/{I}_{L}}$.

The phase difference $\Delta {\varphi }_{{\rm{in}}}$ is retrieved in a single step by the polarization distribution $\left|{{\rm{PD}}}_{{\rm{ring}}}\right\rangle \,$ along the outer ring intentionally added to encircle the Tai Chi Symbol. On the imaging plane, though the phase of each pixel for |*R*〉 is random, the phase difference of the overlapped pixel with another cross-polarization (i.e. |*L*〉) is deterministic. In this case, the phase difference $\Delta \varphi$ of the two images $\left|{R}_{{\rm{TJ}}}\right\rangle \,$ and $\left|{L}_{{\rm{TJ}}}\right\rangle$ along the ring is set as $\Delta \varphi =2\theta$, where $\theta$ is the azimuth angle. Their own phases are ${\varphi }_{R}={\varphi }_{f}-\Delta \varphi /2$ and ${\varphi }_{L}={\varphi }_{f}+\Delta \varphi /2$, where ${\varphi }_{f}$ is the random phase. Therefore, we have:

$$\left|{{\rm{PD}}}_{{\rm{ring}}}\right\rangle ={U}_{R}{e}^{i\left(\Delta {\varphi }_{{\rm{in}}}+{\varphi }_{f}-{\rm{\theta }}\right)}\left|R\right\rangle +{U}_{L}{e}^{i\left({\varphi }_{f}+{\rm{\theta }}\right)}\left|L\right\rangle$$

1 $$={e}^{i\left({\rm{\theta }}+{\varphi }_{f}\right)}({U}_{R}{e}^{i\left(\Delta {\varphi }_{{\rm{in}}}-2\theta \right)}\left|R\right\rangle +{U}_{L}{|L}{{\rangle }})$$

It can be seen that the polarization state is different along the ring though the intensity is uniform when $\left|{\psi }_{s}^{+}\right\rangle$ is incident. Analyzed with a linear analyzer oriented at an angle $\alpha$, the resulted intensity depends on the $\Delta {\varphi }_{{\rm{in}}}$ as follows,

2 $$\begin{array}{l}{I}_{{\rm{ring}}}\left(\alpha ,\theta \right)=\frac{{\left({U}_{R}-{U}_{L}\right)}^{2}}{2}\\\qquad\qquad+2{U}_{R}{U}_{L}{\cos }^{2}(-\alpha -\frac{\Delta {\varphi }_{{\rm{in}}}}{2}+\theta )\end{array}$$

Then ${\Delta \varphi }_{{\rm{in}}}$ can be retrieved by the angle for the maximum intensity along the ring through ${\theta }_{M}=\Delta {\varphi }_{{\rm{in}}}/2+\alpha$. Therefore, the arbitrary incident state $\left|{\psi }_{s}^{+}\right\rangle$ can be reconstructed by extracting ${I}_{R}/{I}_{L}$ and ${\theta }_{M}$ from the single-frame intensity distribution of the vectorial meta-holography after the analyzer.

The state $\left|{\psi }_{s}^{-}\right\rangle ={-a}_{R}\left|{L}_{s}\right\rangle +{a}_{L}{e}^{-i\Delta {\varphi }_{{\rm{in}}}}\left|{R}_{s}\right\rangle$ is orthogonal to $\left|{\psi }_{s}^{+}\right\rangle$ with $\left\langle {\psi }_{s}^{+}|{\psi }_{s}^{-}\right\rangle =0$. When $\left|{\psi }_{s}^{-}\right\rangle$ is incident, we obtain ${a}_{L}/{a}_{R}=\sqrt{{I}_{L}/{I}_{R}}$, $\left|{{\rm{PD}}}_{{\rm{ring}}}\right\rangle ={e}^{i\left({\rm{\theta }}+{\varphi }_{f}-\Delta {\varphi }_{{\rm{in}}}\right)}({-U}_{R}{e}^{i\left(\Delta {\varphi }_{{\rm{in}}}-2\theta \right)}\left|R\right\rangle +{U}_{L}{|L}\rangle )$, and ${I}_{{\rm{ring}}}\left(\alpha ,\theta \right)={\left({U}_{R}+{U}_{L}\right)}^{2}/2-2{U}_{R}{U}_{L}{\cos }^{2}(-\alpha -\Delta {\varphi }_{{\rm{in}}}/2+\theta )$. When the pairs $\left|{\psi }_{s}^{+}\right\rangle$ and $\left|{\psi }_{s}^{-}\right\rangle$ are incident at the same time, the sum of their intensities after the analyzer is$\,{(U}_{R}^{2}+{U}_{L}^{2})/2$ and ${U}_{R}^{2}+{U}_{L}^{2}$ in the Tai Chi Symbol and the ring, respectively. The uniform intensity distribution shows the holographic images when any pairs of cross-polarization states are incident simultaneously.

Using a single photon, the meta-holography with $\left|{\psi }_{s}^{+}\right\rangle$ incidence before and after the analyzer in signal terminal is shown in Fig. 1d. Using entangled photon pairs, the accurate remote control of ECVMH through idler photons, without any prior information, can transport the full-polarization encoded quantum information carried by the signal photons to a distant terminal. In the experiment, a pair of entangled photons ${|\Phi }_{\psi }^{+}\rangle =1/\sqrt{2}(|{\psi }_{s}^{+}{H}_{i}\rangle +|{\psi }_{s}^{-}{V}_{i}\rangle )$ are generated by the spontaneous parametric down-conversion (SPDC), where $|{H}_{i}\rangle$ and $|{V}_{i}\rangle$ are the horizontally and vertically polarized states and subscripts “s” and “i” represent the signal (red) and idler (blue) photons. When the idler photon is measured as $|{H}_{i}\rangle$ or ${{|V}}_{i}\rangle$, the ${|\Phi }_{\psi }^{+}\rangle$ collapses into $\left|{\psi }_{s}^{+}\right\rangle$ or $\left|{\psi }_{s}^{-}\right\rangle$, respectively,

3 $$\left|{\psi }_{s}^{+}\right\rangle =\left\langle {H}_{i}{|\Phi }_{\psi }^{+}\right\rangle, \,\left|{\psi }_{s}^{-}\right\rangle =\left\langle {V}_{i}{|\Phi }_{\psi }^{+}\right\rangle ,$$

By analyzing the holographic image after the analyzer, the polarization of the incident pure state can be reconstructed.

The density matrix of the generated entangled photon-pair is $${\rho }^{{si}}={|\Phi }_{\psi }^{+}\rangle \langle {\Phi }_{\psi }^{+}|$$. We select two eigen states $$\left|{\psi }_{s}^{+}\right\rangle =\left|{L}_{s}\right\rangle \,$$ and $$\left|{\psi }_{s}^{-}\right\rangle =\left|{R}_{s}\right\rangle$$ for the meta-holography design, whose density matrices are $${\rho }_{{\rm{LCP}}}=\left|{L}_{s}\right\rangle \left\langle {L}_{s}\right|$$ and $${\rho }_{{\rm{RCP}}}=|{R}_{s}\rangle \langle {R}_{s}|$$ with $$\left.\left|{\Phi }_{\psi }^{+}\right.\right\rangle$$$$=\left|{\Phi }_{{LR}}^{+}\rangle\right.$$ $$=1/\sqrt{2}(\left|{L}_{s}{H}_{i}\right.\rangle +\left|{R}_{s}{V}_{i}\right.\rangle )$$. When the idler photon is in $$|{H}_{i}\rangle$$ or $$|{V}_{i}\rangle$$ state, the density matrix of signal photon is $${\rho }^{s}={\rho }_{{\rm{LCP}}}$$ or $${\rho }^{s}={\rho }_{{\rm{RCP}}}$$ with both $${{\rm{Tr}}}_{s}[{\rho }^{s}{\rho }^{s}]=1$$, resulting in pure state^40^. The corresponding meta-holographic images of $$\left|{L}_{s}\right\rangle$$ and $$\left|{R}_{s}\right\rangle$$ states are shown in Fig. 1d. When detecting the idler photon and erasing its polarization information, the signal photon collapses into a maximally mixed (MM) state with the density matrix of $${\rho }^{s}={\rho }_{{\rm{MM}}}=(\left|{\psi }_{s}^{+}\right\rangle \langle {\psi }_{s}^{+}|+\left|{\psi }_{s}^{-}\right\rangle \langle {\psi }_{s}^{-}|)/2=(\left|{L}_{s}\right\rangle \langle {L}_{s}|+\left|{R}_{s}\right\rangle \langle {R}_{s}|)/2$$, which only exists in the quantum realm. The MM state is proportional to an identity matrix and is a statistical mixture of two eigen pure states with equal probability. After the analyzer, the meta-holography intensity distribution of MM state in the imaging terminal is different from that with any pure state’s incidence. Due to the characteristic of identity matrix, its intensity can be decomposed of the intensity addition of holographic images when two eigen pure states $$\left|{\psi }_{s}^{+}\right\rangle$$ and $$\left|{\psi }_{s}^{-}\right\rangle$$ with equal probability is incident, in Fig. 1d, we use $$\left|{\psi }_{s}^{+}\right\rangle =\left|{L}_{s}\right\rangle$$ and $$\left|{\psi }_{s}^{-}\right\rangle =\left|{R}_{s}\right\rangle$$.

### Realization of meta-holographic images

**Fig. 2:**
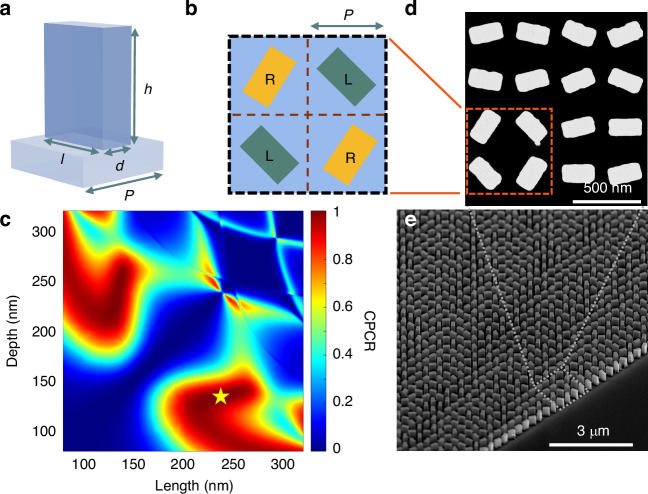
Design of a metasurface for a vectorial metahologram **a, b** Schematic of unit cell and metamolecule of the metasurface. The rectangular silicon pillar in a unit cell is on a sapphire substrate. Each subwavelength metamolecule comprises four unit cells to modulate the phases and amplitudes of LCP (“L”) and RCP (“R”) incident light to generate arbitrary vectorial polarization distribution on the metasurface or the hologram plane. The period $(P)$ and dimensions $\left(h,\,l,\,d\right)\,$ of a unit cell are $(350,\,350,\,240,\,135)$ nm. **c** Calculated CPCR of different unit cells for LCP to RCP conversion. The optimal parameters are marked by the yellow star, with the transmittance of 75% under the 808 nm LCP incidence. **d, e** Scanning electron microscopy images of the fabricated metasurface

We realize arbitrary polarization distribution on the imaging plane using the PB-phase based metahologram with high optical transmission and superior imaging quality, which is essential for the quantum imaging efficiency^33^. As shown in Fig. 2a, the unit cells of the metasurface have identical geometric sizes, easier for fabrication parameter optimization (see Supplementary Information 1). At the same time, we map the phase profiles for LCP and RCP holograms to the metasurface, multiplexing them in an “X” pattern within a metamolecule^41^ in Fig. 2b, and the metahologram comprises 999$\times$999 metamolecules. This approach reduces the crosstalk between polarization channels, provides the suitable FoV and guarantees correct overlap of the reconstructed images to enhance imaging quality (see Supplementary Information 3).

In order to determine vectorial distributions in the far-field, a modified Gerchberg-Saxton (GS) algorithm for the phase retrieval and a typical global optimization algorithm - simulated annealing (SA) are utilized to optimize the phase distribution on the metahologram. In addition, to avoid the influence of the zero-order output on the region of holographic image, we position the designed meta-holography on the upper left of the imaging plane thus its conjugate counterpart locates on the lower right (see Supplementary Information 2 and 3). With optimized parameters from calculated results of CPCR in Fig. 2c, a fabricated sample composed of nearly a million metamolecule pixels is shown in Fig. 2d, e. The high qualities of the holography design and the metasurface fabrication ensure the accurate correspondence of the experimentally generated images with the simulation, leading to the correct reconstruction of the incident polarization state in the quantum realm.

### Multi-channel polarization state reconstruction by the vectorial meta-holography

**Fig. 3:**
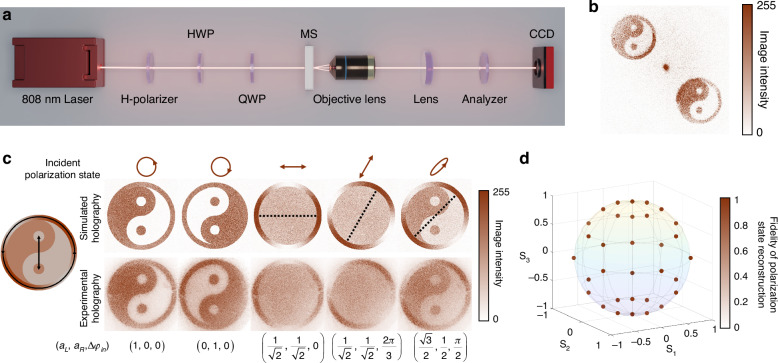
Accurate polarization state reconstruction using the vectorial meta-holography **a** Optical setup for the incident polarization state reconstruction. H-Polarizer, horizontally oriented polarizer; MS, metasurface; CCD, charge coupled device. **b** For LCP incidence, the vectorial meta-holography is in the upper left, and its conjugate is in the lower right. **c** Simulated and experimental holographic images for different polarized states. The images are captured from $({a}_{L},\,{a}_{R},\Delta {\varphi }_{{\rm{in}}})$ of the incident states, and the corresponding polarization ellipse diagrams are as shown above. The angle of ${\theta }_{M}$ is indicated by the dashed line. **d** Experimental fidelities of polarization state reconstruction, defined as the correlation between corresponding incident polarization states $\left|{\psi }_{s}^{+}\right\rangle$ on the Poincaré sphere and the reconstructed states. The reconstructed Stokes parameters $({s}_{0},{s}_{1},\,{s}_{2},\,{s}_{3})$ are presented as the coordinates on the Poincaré sphere^39^

The PB-phase metasurface enables accurate modulation and reconstruction of the corresponding images after the analyzer, and the resulting complementary images ($\left|{R}_{{\rm{TJ}}}\right\rangle \,$ and $\left|{L}_{{\rm{TJ}}}\right\rangle$) are sensitive to incident polarization states. This is exploited for accurate reconstruction of polarization states from holographic images (see Supplementary Information 2). We extract the incident state $\left|{\psi }_{s}^{+}\right\rangle$ by acquiring the amplitude ratio $\sqrt{{I}_{R}/{I}_{L}}$ from the Tai Chi Symbol and the orientation angle ${\theta }_{M}$ from the ring, respectively. ${I}_{R}\,$ and ${I}_{L}$ are obtained by averaging the intensity distribution of $\left|{R}_{{\rm{TJ}}}\right\rangle \,$ and $\left|{L}_{{\rm{TJ}}}\right\rangle$ in Tai Chi Symbol. By fitting the function of intensity distribution along the ring, ${\theta }_{M}$ is obtained as described in Eq. (2). By collecting the intensities of all pixels after the analyzer, this method minimizes the measurement error due to the fabrication deviation or intensity fluctuation of the individual pixel and improves the capability of polarization measurement^42^. To reduce the interference patterns arising from the reflections between the objective lens and the metasurface, the angle $\alpha$ of the analyzer in Fig. 3a is slightly adjusted 2° away from 0°.

To generate arbitrary $\left|{\psi }_{s}^{+}\right\rangle$, a combination of a half-wave plate (HWP) and a quarter-wave plate (QWP) is used. A typical meta-holography of $\left|{R}_{{\rm{TJ}}}\right\rangle$ is shown in Fig. 3b. By rotating the HWP or QWP from the horizontal direction at a step of 15°, 32 discrete data sets in total are acquired. As presented in Fig. 3c, the experimental results for LCP, RCP, two linearly polarized (LP) and an elliptical polarization state are consistent with the simulated holography. The number of imaging channels is the maximum in the quantum domain for reconstructing arbitrary pure and maximally mixed states^3^. After mapping the extracted polarization states characterized by Stokes parameters $({s}_{0},\,{s}_{1},\,{s}_{2},\,{s}_{3})$ onto the Poincaré sphere with ${s}_{0}=1$ namely ${a}_{L}^{2}+{a}_{R}^{2}=1$, the average fidelity is higher than 94.78% ± 3.04%, with a maximum of 99.52% and a minimum of 88.84%, as shown in Fig. 3d. Particularly, the average fidelity of the LP states at the equator and circularly polarized states at poles is higher than 96% (see Supplementary Information 4). By capturing a single-frame holographic image, the polarization state of the signal photon is reconstructed and the idler photon state is concurrently revealed through the quantum entanglement. In other words, this method realizes the projection measurement of multiple pairs of orthogonal bases simultaneously, thus simplifying multiple measurements in quantum state reconstruction. In the meantime, the high reconstruction fidelities for multiple states verify the ability of high SNR multi-channel holographic images by remote control of the entanglement states.

### Remote control of entanglement-based vectorial meta-holography with a high SNR

**Fig. 4:**
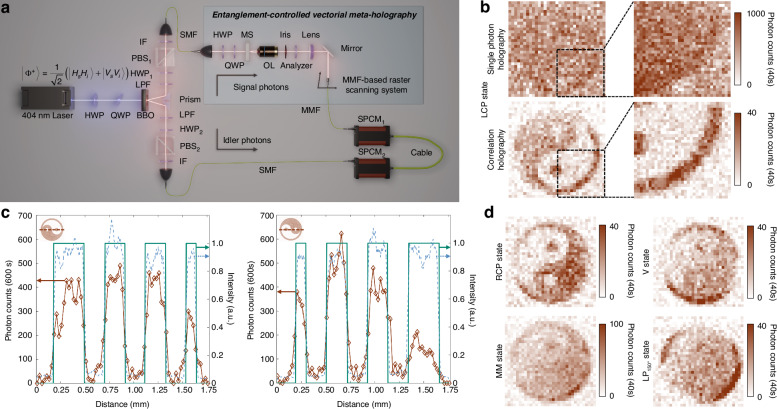
Experimental characterization of the entanglement-controlled vectorial meta-holography (ECVMH) **a** ECVMH characterization setup. LPF, long-pass filter; PBS, polarizing beam splitter; IF, interference filter; SMF, single-mode fiber; MMF, multi-mode fiber. **b** The SNR enhancement from single photon (upper) to correlation (lower) holography, calculated as ${\rm{SNR}}=10\log ({I}_{\rm{signal}}/{I}_{\rm{background}})$. **c** The photon counts and intensity along a line across the center of the Tai Chi Symbol. The comparison of the experimental photon counts (red diamond), simulated intensity (blue dashed line), and ideal intensity (green line) for ECVMH ($\left|{R}_{{\rm{TJ}}}\right\rangle \,$ and $\left|{L}_{{\rm{TJ}}}\right\rangle$) is shown by scanning a line across the center of Tai Chi Symbol, corresponding to $|{L}_{s}\rangle$ and $|{R}_{s}\rangle$ states, respectively. The upper left insets display the region scanned from left to right. **d** Multiple ECVMH results for pure and MM states incidence. The ECVMH acquired from pure (${|R}_{s}\rangle$, $|{V}_{s}\rangle$, and $|L{P}_{150^{\circ} ,s}\rangle$) and MM states when incident states are triggered to $|{V}_{i}\rangle$ and MM states, respectively

Under dim light, quantum holographic images are usually obscured by the environmental noise, even though the state reconstruction fidelity is high for classical light. With the help of coincidence measurements between entangled photons, the second-order optical coherence significantly enhances the imaging SNR by effectively blocking background noise. This enhancement is demonstrated by the value of the upper limit ${\rm{SNR}}=10\log \bar{{I}_{s}^{b}}/{C}_{{\rm{accidental}}}$, where $\bar{{I}_{s}^{b}}$ is the average noise within a time bin in the signal terminal, and ${C}_{{\rm{accidental}}}$ is the accidental coincidences (see Supplementary Information 8). In our experiment as shown in Fig. 4a, a continuous laser with central wavelength of 404 nm passes through a $\beta$-barium borate (BBO) crystal to generate the entangled states $\left|{\Phi }^{+}\right.\rangle =1/\sqrt{2}(\left|{H}_{s}{H}_{i}\right.\rangle +\left|{V}_{s}{V}_{i}\right.\rangle )$. The signal photon is directed into the holographic imaging path and then captured by a multi-mode fiber (MMF)-based raster scanning system. Using the MMF, the coupling and transmission efficiency are preserved, and the induced mode mixing and the bending loss of the MMF have little impact on the quantum imaging. Due to the smaller numerical aperture and the use of motorized translation stages, we achieve lower noise, leading to higher imaging efficiency and resistance to stray light compared to similar results by using a camera (see Supplementary Information 8)^28,31,32,37^, while the idler photon is measured by a detection system consisting of an HWP, a PBS, and a single photon count module (SPCM).

The quantum entanglement of $\left|{\Phi }^{+}\right.\rangle$ is preserved after the metasurface to ensure the realization of the ECVMH. Using the measurement of the S parameters, quantum state tomography (QST), and two-photon polarization interference fringes (see Supplementary Information 7), we perform a Bell test using Clauser-Horne-Shimony-Holt (CHSH) inequality^43^ and reconstruct the density matrix using maximum likelihood estimation method^44^ by recording coincidence counts between the idler and zero-order photons. The zero-order photons retain the original wavefronts of the incident light^45^. The obtained CHSH value is $S=2.644\pm 0.058$ with fidelity of $F=0.929\pm 0.006$, close to those for the original SPDC source with $S=2.703\pm 0.019$ and $F=0.933\pm 0.002$. The Bell-type violation ($S\ge 2$) and near-unity fidelity confirm that the entanglement preservation after the metasurface and the reconstructed state is close to the desired state $|{\Phi }^{+}\rangle$.

For demonstrating the eigen states $|{L}_{s}\rangle \,$ and $|{R}_{s}\rangle$ in our design, the signal polarization state $|{\Phi }^{+}\rangle$ is converted to $\left|{\Phi }_{{LR}}^{+}\right.\rangle$ in our experiment. As indicated in Eq. (3), when the idler photon is triggered to $|{H}_{i}\rangle$ and $|{V}_{i}\rangle$, the holographic images are $\left|{R}_{{\rm{TJ}}}\right\rangle \,$ and $\left|{L}_{{\rm{TJ}}}\right\rangle$, as shown in Fig. 4b, d. Because the MM quantum state is the uniform mixture rather than the coherent superposition of a pair of cross-polarization states, its corresponding image is different from that for a pure quantum state or the classical light. When detecting the idler photon and erasing its polarization information, the intensity of the holographic image with MM state incidence in the signal terminal is the sum of the intensities with $\left|{R}_{{\rm{TJ}}}\right\rangle \,$ and $\left|{L}_{{\rm{TJ}}}\right\rangle$ incidence, as shown in Fig. 4d.

ECVMH can markedly enhance imaging SNR of the correlation holography by controlling the state of idler photons, as demonstrated in the following results. In our experiment, the signal photon collapses into $|{L}_{s}\rangle$ or MM state by setting HWP_2_ to 0° or removing PBS_2_ in the idler terminal, respectively. Using the raster scanning system to detect the signal photon, the ECVMH is acquired with 40 s integration time, 50 μm scanning step and 33 steps, the total integration time is approximately 12.6 h, without HWP_1_ and PBS_1_. In Fig. 4b, the retrieved correlation holography is clear with a high-contrast ring but the one using single photon is blurry. When the idler photon is triggered to $|{H}_{i}\rangle \,$ or $|{V}_{i}\rangle$ with $\left.\left|{\Phi }_{{LR}}^{+}\right.\right\rangle$ incidence, the coincidence photon counts in a line across the center of the Tai Chi Symbol are shown in Fig. 4c, with 600 s integration time, 25 μm scanning step and 71 steps, and the total integration time is approximately 11.8 h. The ECVMH for ${|L}_{s}\rangle$ state incidence exhibits high SNR of 10.78 dB compared to 0.72 dB in the single photon holography, and the former is closed to the simulated SNR of 11.69 dB for the LCP results (see Supplementary Information 8). The obtained intensity in red approaches the simulated one in blue, while the ideal scenario is presented in green. Similar results for RCP are given at the right side, where the simulated and experimental SNR are 10.87 dB and 9.08 dB, with SNR of 0.01 dB in the single photon holography. With a longer integration time, the SNR enhancement will be close to the estimation as shown in Supplementary Information 8.

Meanwhile, the high imaging SNR enhancement leads to the high fidelity reconstruction of the incident polarization state from the entanglement-controlled vectorial holographic images. When the signal photon collapses into $|{R}_{s}\rangle$ and MM states, the corresponding correlation holography is correctly reconstructed as shown on the left of Fig. 4d. For the MM state incidence, the intensity of the ECVMH after the analyzer is uniform in both the Tai Chi Symbol and the ring, respectively. For an arbitrary linear polarization state $\left|{\psi }_{s}^{+}\right\rangle$ with superposition of ${|L}_{s}\rangle$ and ${|R}_{s}\rangle$, like $\left|{V}_{s}\right\rangle ,\,|L{P}_{150^{\circ} ,s}\rangle$ on the right, the angle ${\theta }_{M}$ of correlation holography is $\Delta {\varphi }_{{\rm{in}}}/2=\pi /2,\,5\pi /6$, respectively. The fidelity of reconstructed polarization states is 83.38%, 92.77%, 83.91% and 88.21% for ${|L}_{s}\rangle$, ${|R}_{s}\rangle$, $|{V}_{s}\rangle$, and $|L{P}_{150^{\circ} ,s}\rangle$, respectively, and the average fidelity is 87.07% ± 4.37%.

As demonstrated above, the proposed entanglement-controlled vectorial meta-holography by multiplexing two cross-polarized states eliminates the need for an additional detector and reconstructs the incident polarization state in a single step. By further enhancing the imaging quality of meta-holography and transmission efficiency of metasurface, higher reconstruction fidelity can be achieved. One of its potential applications in the quantum realm is for QST, which is a widely used method to characterize multiphoton entanglement states^46^. The PB phase-based meta-holography design is robust in fabrication and assembly without pre-polarization calibration for structural fabrication errors, providing a potential route for high accuracy and efficiency of QST, to realize an inexpensive, compact, and fully automated system^47^. Moreover, quantum holography has been utilized in remotely controllable imaging^37^ and cryptography^48^, so the entanglement-controlled vectorial meta-holography integrated with photonic circuits^49–51^ with multiple degrees of freedom (e.g., polarization, frequency, and OAM) can facilitate the manipulation of the quantum light field and further miniaturization of optical system.

## Discussion

In summary, we have proposed and experimentally demonstrated the entanglement-controlled vectorial meta-holography. Using a high transmittance dielectric metasurface, this novel quantum holography precisely manipulates the phase, amplitude, and polarization on the imaging plane. It achieves high-fidelity reconstruction of amplitude ratio and phase difference of incident polarization states and enables remote control of holographic images with high imaging SNR. The powerful capability of polarization state reconstruction is demonstrated as the identification of 32 imaging channels with an average fidelity up to 94.78%. Using entangled idler photons to remotely control the holographic images reconstructed by the signal photons, the imaging SNR is 10.78 dB. Additionally, the use of an MMF-based raster scanning system reduces the reliance on bulky and expensive quantum imaging cameras.

Using additional independent photonic degrees of freedom, such as incident angles and OAM modes, meta-holography allows more independent imaging channels. The image quality can be enhanced by using brighter quantum sources. To further improve the imaging speed, the fast micro-electromechanical systems can be used to replace the slow mechanical moving components. With these developments, the entanglement-controlled vectorial meta-holography will find potential applications in quantum information processing.

## Materials and methods

### Numerical simulation

The finite element simulation (COMSOL) is applied to simulate a mono-crystalline silicon rectangular pillar in a unit cell on a sapphire substrate. The utilized periodic boundary condition (PBC) and perfect matched layers (PML) are along transverse and longitudinal corresponding to the propagation of the incident light. The incident and transmitted lights are excited and measured with port boundary conditions. Due to the PB phase-based metasurface being composed of HWP for each unit cell, we use CPCR = 1 as the evaluation metric.

### Sample fabrication

A lift-off process is employed for the fabrication. Firstly, an 80 nm thick positive resist (AR-P 6200.04, Allresist) is spin-coated at 4000 rpm for 1 min onto a 350 nm thick mono-crystalline silicon film (crystal orientation: [100]) on a sapphire substrate (R plane), cleaning with acetone and isopropanol using the ultrasound; then the positive resist is baked at 150 °C for 1 min. Subsequently, the positive resist is exposed to 20 kV electron beam lithography (EBL, Raith E-line Plus) for ~60 min, with the optimal dose for individual structures of 43 μC/cm^2^. Following the exposure, the exposed positive resist is developed by the developer (AR 500–546) for 1 min, and the exposed pattern is fixed with isopropanol for 30 s, then the pattern is rinsed with deionized (DI) water for 30 s. Afterward, a 30 nm thick layer of chromium (Cr) is deposited on the exposed surface as a hard mask using electron beam evaporation (EBE, DE500C, DETECH) for ~30 min, then the Cr-layer in the unexposed region is removed out using the AR 600-71 remover. The sample is etched using inductively coupled plasma (ICP) for ~5 min. Then the hard mask is removed by chromium etchant solution containing cerium ammonium nitrate (CAN) and nitric acid (HNO_3_) for 1 min.

### Optical setup for polarization state reconstruction

Since the incidence of classical light and single photon yield the same results, the LP light is generated by a supercontinuum laser to facilitate experimental testing. The 808 nm light generated by the supercontinuum laser (FIU-15, NKT Photonics) passes through a horizontally oriented analyzer. It then goes through a wave plate group (combining an HWP and a QWP) to generate arbitrary polarization states, and it is subsequently incident on the $0.7{{\times }}0.7$ mm^2^ metasurface. The outgoing light from the metasurface is collected through a 40× objective lens (NA 0.75, Olympus) and transmits to a 50 mm lens and a linear analyzer then to the CCD (CS165CU1, Thorlabs) imaging plane for capturing meta-holography.

### Optical setup for ECVMH

A continuous 50 mW LP light with wavelength of 404 nm is generated using an external-cavity diode laser (ECL801, UniQuanta). After polarization adjustment by a 404 nm HWP and a 404 nm tilted QWP, it is directed into a BBO crystal to induce Type-I SPDC generating pairs of 808 nm photons with quantum entanglement. These pairs are reflected into two symmetric optical paths using small-angle prism and pass through 650 nm LPF, 808 nm HWP, PBS, then 3 nm IF centered at 808 nm. The pair of HWP and PBS is used for polarization measurement. They are coupled to fiber lenses and the idler photon is received by a SPCM_2_ (SPCM-850-14-FC, Excelitas) via SMF (780-HP) after transmission. The signal photon, following the SMF, exits via a fiber coupler, passes through the wave plate group then enters the metasurface. The outgoing light is collected by the imaging path above using the raster scanning system in lieu of the CCD. Using raster scanning system for receiving the signal photon and transmitting to SPCM_1_ (SPCM-850-14-FC), it enables the measurement of single photon and correlation holography by transferring both SPCMs to a time-to-digital converter (Time Tagger Ultra, Swabian Instruments). The raster scanning system consists of a 50 μm core diameter MMF (MMC50L-0.22-PC-2, LBTEK) coupled with two orthogonal fixed motorized linear translation stages (NRT150/M, Thorlabs) controlled by an integrated homemade LabVIEW (National Instruments) program. All optical fibers are coated with an 808 nm anti-reflection (AR) film to improve optical transmittance.

## Supplementary information

Supplementary Information for: Entanglement-Controlled Vectorial Meta-Holography

## Data Availability

All data needed to evaluate the conclusions in this paper are present in the paper and/or the Supplementary Information.
