# Supplementary material for: Entanglement-controlled vectorial meta-holography
Source: Light Sci Appl. 2025 Mar 25;14:135. doi: 10.1038/s41377-025-01818-w (PMC11937580; doi:10.1038/s41377-025-01818-w)
Supplement: Supplementary file 1 — Supplementary Information for: Entanglement-Controlled Vectorial Meta-Holography [file 41377_2025_1818_MOESM1_ESM.pdf]

Supplementary Information for:
Entanglement-Controlled Vectorial Meta-Holography

Sheng Ye[1], Yue Han[1], Li-Zheng Liu[1], Weiping Wan[1], Ruiqi Wang[1], Mingna Xun[1], Qiang Li[1], Qihuang Gong[1,2,3,4,5], Jianwei Wang[1,2,3,4,5], Yan Li[1,2,3,4,5,⋆]

[1] *State Key Laboratory for Artificial Microstructure and Mesoscopic Physics, School of Physics, Peking University, Beijing, 100871, China.*

[2] *Frontiers Science Center for Nano-optoelectronics & Collaborative Innovation Center of Quantum Matter, Peking University, Beijing, 100871, China.*

[3] *Collaborative Innovation Center of Extreme Optics, Shanxi University, Taiyuan 030006, Shanxi, China.*

[4] *Peking University Yangtze Delta Institute of Optoelectronics, Nantong 226010, Jiangsu, China.*

[5] *Hefei National Laboratory, Hefei 230088, China.*

⋆*Corresponding author: li@pku.edu.cn*

Contents

1. Details of sample fabrication **2**

2. The design and simulation of vectorial meta-holography **2**

3. Field of view of vectorial meta-holography **4**

4. Polarization state reconstruction from the vectorial meta-holography **5**

5. Determining the ϕ_q from the spontaneous parametric down-conversion (SPDC) **6**

6. Comparison of imaging by using raster scanning system and electron-multiplying charge coupled device **7**

7. The entanglement correlation of SPDC source and the zero-order output **8**

8. The imaging SNR of single photon holography and correlation holography **9**

9. The conversion between two quantum entanglement states **11**

1. Details of sample fabrication

The processing flowchart is outlined in Fig. S1 and we improve the precision of metasurface fabrication in the electron beam lithography (EBL) exposure by reducing the spin-coating layer thickness and lowering the development temperature. One of the most important aspects of the processing procedure is the EBL. The real exposure time of the corners in the rectangle nano-blocks is shorter than the setting exposure time inside of the rectangular region, causing the "*fillet*" effect. The proximity effect correction (PEC) method has been used to ease this problem. To achieve the high-quality rectangular patterns, the exposed positive resist is developed at a lower temperature and deposited by a thinner Cr-layer. The EBE and ICP etching are also challenging aspects because of the complexity of the simultaneous optimization of many fabrication parameters: the etching dose, processing recipe, processing time and environmental parameters (temperature, humidity). The fabrication quality is often evaluated from the dimensions of nano-blocks by capturing the SEM images.

The advantage of Pancharatnam-Berry (PB) phase-based metasurface lies in their broad spectral response and high transmission efficiency, along with consistent geometric sizes, making the fabrication easier. We adopt the procedure in Materials and Methods section in the main text and improve the precision of the metasurface fabrication in EBL with thinner spin-coated layer and lower development temperature. Additionally, we enhance the accuracy of pattern transfer by reducing the thickness of the chromium mask. To ensure process repeatability, scanning electron microscopy (SEM) characterization is performed after each essential step. Also, we develop an SEM image recognition program based on the Hough transform[S1] to measure the angles and size of the rectangular structures on the metasurface. By iteratively optimizing the processing procedure, we realize the manufacture of the high-quality nanostructures, where the angular error and structure size variance are less than 1.5° and 2.5%.

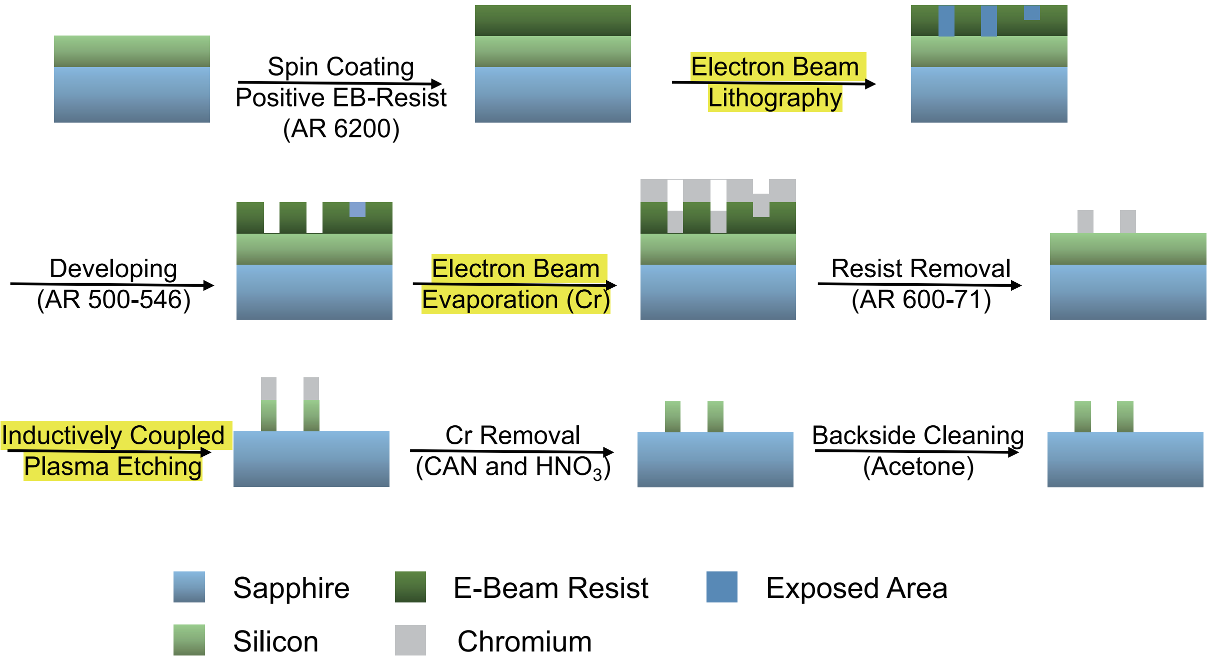

Fig. S1 The processing flowchart of the metasurface.

2. The design and simulation of vectorial meta-holography

To achieve the ability to produce arbitrary polarization distributions in the far-field, the sum of the intensities after the analyzer for designed vectorial holographic images is equal when any polarization state is incident. Fig. S2a depicts the holographic images on the imaging plane after the incidence with superposition of left (LCP) and right circularly polarized (RCP) states.

The two images both features an inner Tai Chi Symbol and an outer ring in saddle red representing $|R_{\mathrm{TJ}}\rangle = U_R e^{i\varphi_R}|R\rangle$ and $|L_{\mathrm{TJ}}\rangle = U_L e^{i\varphi_L}|L\rangle$ for LCP and RCP incidence, respectively. Their amplitudes are binary distributed, while φ_L and φ_R are randomly distributed in the far-field. When only $|L_s\rangle$ or $|R_s\rangle$ component is incident on the PB-phase based metasurface, it is converted into the Tai Chi Symbol with RCP or LCP state, accompanied by an unconverted zero-order output retaining LCP or RCP state. Because the cross-polarization conversion ratios for $|L_s\rangle$ or $|R_s\rangle$ state are identical and the bright area of both $|R_{\mathrm{TJ}}\rangle$ or $|L_{\mathrm{TJ}}\rangle$ images are equal. Their power and the average intensities I_R and I_L of the red area in the inner Tai Chi Symbol region are proportional to the incident intensities, leading to $a_L/a_R = \sqrt{I_R/I_L}$. After the polarizer, the average intensities will be $U_L^2/2$ and $U_R^2/2$, respectively.

The arbitrary incidence state can be decomposed into $|\psi_s^+\rangle = a_L e^{i\Delta\varphi_{\mathrm{in}}}|L_s\rangle + a_R|R_s\rangle$. Additionally, a phase difference $\Delta\varphi = \Delta\varphi_{\mathrm{ring}} = 2\theta$ ($\theta \in [0, 2\pi)$) with the steel blue in the ring is set between the $|R_{\mathrm{TJ}}\rangle = e^{i(\varphi_f - \Delta\varphi/2)}|R\rangle$ and $|L_{\mathrm{TJ}}\rangle = e^{i(\varphi_f + \Delta\varphi/2)}|L\rangle$ with same binary amplitude on the imaging plane. When $|\psi_s^+\rangle$ state is incident, the polarization distribution $|\mathrm{PD}_{\mathrm{ring}}(\theta)\rangle$ along the ring is,

$$\begin{aligned}|\mathrm{PD}_{\mathrm{ring}}(\theta)\rangle &= U_R e^{i\Delta\varphi_{\mathrm{in}}} e^{i(\varphi_f - \Delta\varphi/2)}|R\rangle + U_L e^{i(\varphi_f + \Delta\varphi/2)}|L\rangle \\ &= U_L e^{i(\varphi_f + \Delta\varphi_{\mathrm{in}}/2)}(\sqrt{\tilde{\eta}} e^{i\tilde{\varphi}}|R\rangle + e^{-i\tilde{\varphi}}|L\rangle), \text{ where } \tilde{\varphi} = (\Delta\varphi_{\mathrm{in}} - \Delta\varphi)/2, \tilde{\eta} = (U_R/U_L)^2.\end{aligned}$$

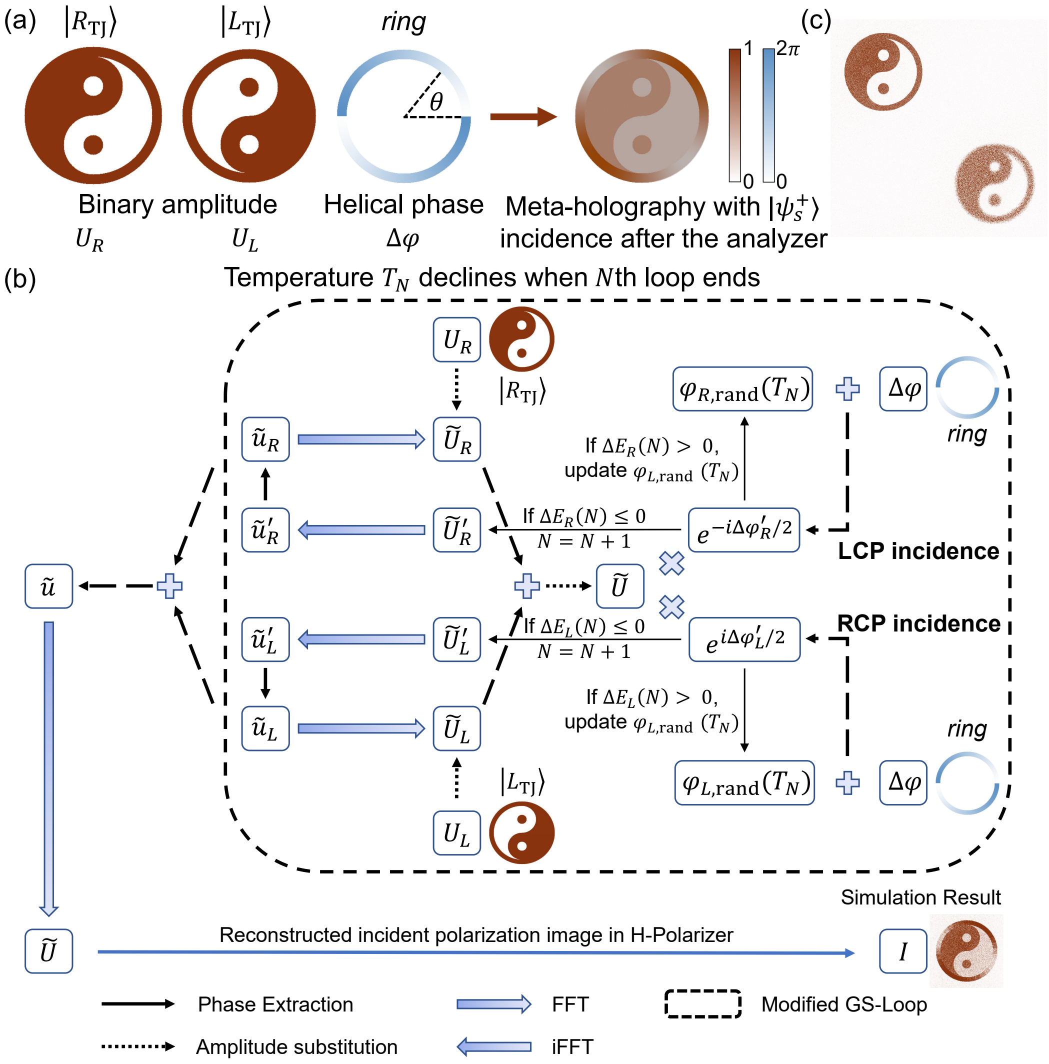

Fig. S2 The design principle and simulation. **a,** Design principle to generate vectorial meta-holography. The $|R_{\mathrm{TJ}}\rangle$ and $|L_{\mathrm{TJ}}\rangle$ contains an inner Tai Chi Symbol and an outer ring designed for LCP and RCP incidence, respectively. The incident amplitudes are binary, and its phase difference of the $\Delta\varphi_{\mathrm{in}}$ are set to 0-2π. After a horizontally oriented analyzer, the meta-holography exhibits spatially distinct distributions of intensity. **b,** Schematic of the modified GS algorithm combined with SA. **c,** Simulated mete-holography with LCP incidence.

After an angle of α oriented polarizer $|P\rangle = \cos\alpha\,|H\rangle + \sin\alpha\,|V\rangle$, according to Malüs's Law $I(\alpha) = I_0\cos^2(\alpha)$, the corresponding intensity along the ring will be,

$$\begin{aligned}
I_{\mathrm{ring}}(\alpha,\theta) &= |\langle P|\,\mathrm{PD}_{\mathrm{ring}}\rangle|^2 = |U_L(\sqrt{\tilde{\eta}}e^{i\tilde{\varphi}}\langle P\,|R\rangle + e^{-i\tilde{\varphi}}\langle P\,|L\rangle)|^2 \\
&= |U_L(\sqrt{\tilde{\eta}}e^{i\tilde{\varphi}}e^{i\alpha} + e^{-i\tilde{\varphi}}e^{-i\alpha})|^2/2 \\
&= (U_R - U_L)^2/2 + 2U_RU_L\cos^2(-\alpha - \Delta\varphi_{\mathrm{in}}/2 + \theta),
\end{aligned}$$

where the LCP (RCP) state is a superposition of $|H\rangle$ and $|V\rangle$, defined as $|L\rangle = \frac{1}{\sqrt{2}}(|H\rangle - i\,|V\rangle)$ and $|R_s\rangle = \frac{1}{\sqrt{2}}(|H\rangle + i\,|V\rangle)$. Then $\Delta\varphi_{\mathrm{in}}$ can be retrieved by the angle for the maximum intensity along the ring through $\theta_M = \Delta\varphi_{\mathrm{in}}/2 + \alpha$. In particular, when $a_L = a_R$, the polarization states is linear polarization (LP) state along the ring, with its polarization angle varying with the azimuth angle θ.

The light modulation of a metasurface's unit cell, which acts as a π-phase retarder, can be represented as Jones matrix. Four unit cells combine to form a subwavelength metamolecule, each with a different rotation angle Θ_i ($i = L, R$), corresponding to "L" and "R" in main text Fig. 2b. The incident $|H_s\rangle$ and $|V_s\rangle$ undergoes modulation by the Jones matrix J_i, resulting in an output state written as:

$$\begin{pmatrix} H_s \\ V_s \end{pmatrix}_{\mathrm{out}} = J_i \begin{pmatrix} H_s \\ V_s \end{pmatrix}_{\mathrm{in}} = \begin{pmatrix} \cos\Theta_i & -\sin\Theta_i \\ \sin\Theta_i & \cos\Theta_i \end{pmatrix} \begin{pmatrix} e^{i\pi} & 0 \\ 0 & 1 \end{pmatrix} \begin{pmatrix} \cos\Theta_i & \sin\Theta_i \\ -\sin\Theta_i & \cos\Theta_i \end{pmatrix} \begin{pmatrix} H_s \\ V_s \end{pmatrix}_{\mathrm{in}}.$$

Since the metamolecule is smaller than one wavelength, the complex amplitude modulation of the metamolecule is the sum of phase modulation of the unit cell for "L" and "R" structure. The output using the circular polarization states can be expressed as:

$$\begin{pmatrix} L_s \\ R_s \end{pmatrix}_{\mathrm{out}} = \begin{pmatrix} 0 & \cos(\Theta_L - \Theta_R)e^{i(\Theta_L+\Theta_R)} \\ \cos(\Theta_L - \Theta_R)e^{-i(\Theta_L+\Theta_R)} & 0 \end{pmatrix} \begin{pmatrix} L_s \\ R_s \end{pmatrix}_{\mathrm{in}} = \begin{pmatrix} 0 & \cos\Theta_A e^{i\Theta_D} \\ \cos\Theta_A e^{-i\Theta_D} & 0 \end{pmatrix} \begin{pmatrix} L_s \\ R_s \end{pmatrix}_{\mathrm{in}},$$

where $\Theta_D = \Theta_L + \Theta_R$ and $\Theta_A = \Theta_L - \Theta_R$ are to the diagonal (D) and anti-diagonal (A) angles between Θ_L and Θ_R. In this way, the metamolecule can modulate both the phase (Θ_D) and amplitude ($\cos\Theta_A$) of the incident state, which are so-called double-phase method[S2].

To achieve high-quality vectorial holography, we employ a modified Gerchberg-Saxton (GS) algorithm for the phase retrieval and a typical global optimization algorithm - simulated annealing (SA) to optimize the phase distribution on the metahologram, resulting in the desired holographic images, as shown in Fig. S2b. The SA is inspired by the principles of solid-state annealing, whose loop starts at a relatively high initial temperature T_0 and gradually decreases the temperature T until it reaches a level that satisfies the thermal equilibrium condition (thus optimize the energy E to global minimum). At each T_N, several rounds of searching are performed. In each round, the method is introduced the random perturbations to the current solution to generate a new solution, and accept the solution according to specific rules, then decreases the temperature to T_{N+1}. The GS algorithm sometimes is trapped in a local minimum, known as the phase stagnation[S3], the combination of the simulated annealing algorithm helps to eliminate phase stagnation and enhance imaging quality by introducing random perturbations and lowering the temperature gradually[S4]. During high temperatures, we use an exponential decay factor; and at lower ones, a Cauchy decay factor is employed, allowing for enough perturbations even in this condition. Here, the sum of squared errors (SSE) is used to measure the similarity between theoretical and simulated holographic images ($\text{SSE}_\sigma = \sum_{ij}(|\widetilde{U}'_{\sigma,ij}| - U_{\sigma,ij})^2 / \sum_{ij} U^2_{\sigma,ij}$); as the Nth of iterations changes, the change of SSE is denoted as $\Delta E_\sigma(N) = \text{SSE}_\sigma(N+1) - \text{SSE}_\sigma(N)$, where $\sigma = L(R)$ for LCP (RCP) incidence.

The GS iteration workflow is as follows: firstly, randomly generate the initial phase distributions $\widetilde{u}_R$ ($\widetilde{u}_L$) on the metahologram. After applying fast Fourier transform (FFT), transform them into far-field amplitudes $\widetilde{U}_R$ ($\widetilde{U}_L$) and replace them with the amplitudes of the preset images U_R (U_L). Sum the $\widetilde{U}_R$ and $\widetilde{U}_L$, then add the preset phase difference $\Delta\varphi$ with a random phase distribution $\varphi_{\text{rand}}(T_N)$ related to the temperature of the SA as a perturbation. This phase is respectively mapped onto the LCP (RCP), resulting in complex amplitudes $\widetilde{U}'_R$ ($\widetilde{U}'_L$).

At this point, check whether $\text{SSE}_\sigma(N+1)$ is smaller than $\text{SSE}_\sigma(N)$, which means $\Delta E_\sigma(N) \leq 0$. If it is true, regenerate the corresponding random phase and recalculate; otherwise, continue the process (T_N will decrease as the number of iteration loops N increases). After applying iFFT (inverse fast Fourier transform), obtain the intensity of the metahologram in PB phase (note that the PB phase-designed metasurface will change the output chiral of polarization states), extract the phases to use as $\widetilde{u}'_R$ ($\widetilde{u}'_L$) for the next iteration. Then repeat for N times.

Finally, extract the phase of $\widetilde{u}_R$ ($\widetilde{u}_L$) after N iterations then calculate the Fresnel diffraction. Under the incidence $|\psi_s^+\rangle$, the holographic image is obtained by filtering its horizontal component. Fig. S2c presents the simulated holography based on Fresnel diffraction appears in the upper left of the image plane, while its conjugate image is in the lower right (clear at the distance of $-L$, see Supplementary Information 3), forming a centrally symmetric image. By employing less speckle noise design of computer generated holography (CGH) with compressed sensing (CS) imaging algorithms, it is possible to obtain holographic images using fewer time and improve the measurement accuracy of reconstructed images[S5,S6].

3. Field of view of vectorial meta-holography

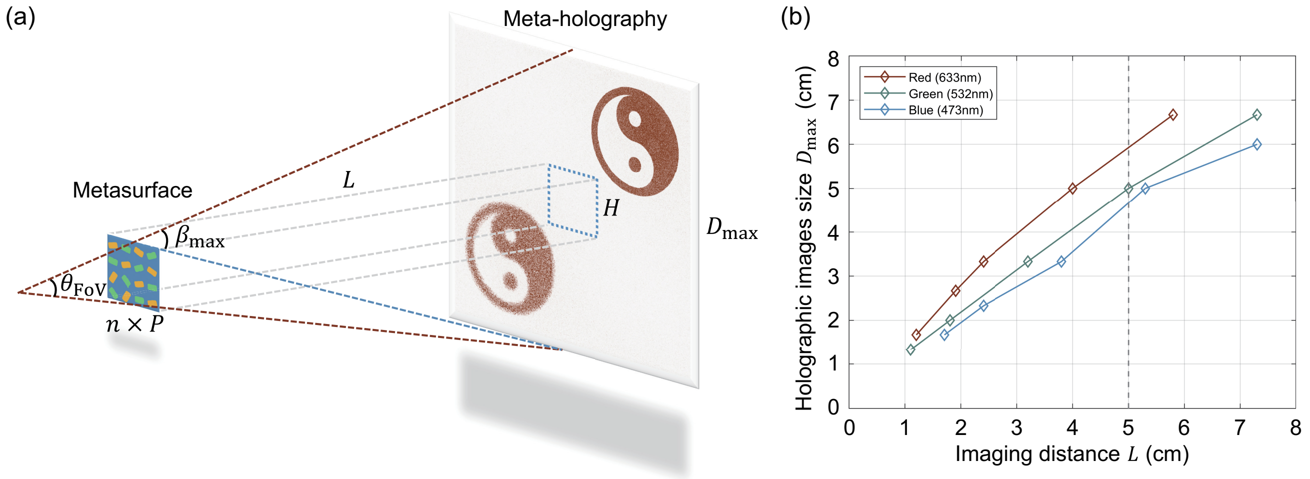

Fig. S3 Analysis of the field of view. a, Schematic of the principle of FoV. After passing through the metasurface, the incident light undergoes diffraction. Its corresponding maximum diffraction angle at each point determines the meta-holography size in the far-field. **b,** The relationship between the size of holographic images and the imaging distance for different visible wavelengths. These wavelengths include red (633 nm), green (532 nm), and blue (473 nm) generated by a supercontinuum laser. The dashed line represents the comparison of holographic size when the imaging plane is set at 5 cm from the metasurface.

When designing holographic images, it is important to consider the field of view (FoV). A too large FoV can make it difficult for the light field to be collected by objective lens (OL) and finally imaged on the camera. After the metasurface, the light propagates a distance L, and forms meta-holography in the far-field, shown in Fig. S3a. Considering the Fresnel diffraction, the maximum diffraction angle from metasurface can be defined as $\beta_{\max} = 2\tan^{-1}(1.43\lambda/\epsilon)$, where λ is wavelength and ϵ is spatial frequency of the phase sampling (with the largest value equal to twice the period P of the phase pixels, $\epsilon \approx 2P$)[S7]. Therefore, the maximum size of holographic image is:

$$D_{\max} = 2L \cdot \tan\frac{\beta_{\max}}{2} - H,$$

where L corresponds to the distance from the metasurface (hologram plane) to the meta-holography (holographic imaging plane), and $H = n \times P$ represents the size of the metahologram (n is the number of metamolecules). Based on the analysis of Fresnel diffraction by rectangular apertures, the FoV (θ_{FoV}) can be expressed as:

$$\theta_{\mathrm{FoV}} = 2\tan^{-1}\frac{D_{\max}}{2L} = 2\tan^{-1}\left(\frac{1.43\lambda}{2P} - \frac{nP}{2L}\right).$$

As shown in the far-field, the rectangular period P increases, and the FoV decreases drastically. We place the imaging plane at a distance of $L = 5$ cm, where $nP/2L \approx 0$; then $\theta_{\mathrm{FoV}} \approx \beta_{\max}$. When the wavelength λ is 808 nm, the calculated θ_{FoV} based on $P = 350$ nm and $n = 999$ pixels is: $\theta_{\mathrm{FoV}} = 117.6°$. A too large FoV is not conducive to the collection of light by the objective lens. Therefore, a multiplexed "X" pattern is adopted, doubling the period in both directions, so that $\theta_{\mathrm{FoV}} = 78.6°$.

In Fig. S3b, we measure the relationship between the sizes of the meta-holography and the distances for sets of visible lights (red, green, and blue). According to the calculation and measurement, the FoVs are as follows: Red (65.2°, 61.3 ± 2.5°), Green (56.4°, 53.1° ± 2.7°), Blue (50.9°, 50.1° ± 2.8°). Therefore, we extrapolate the measured FoV for invisible 808 nm, which are close to the calculation.

At the same time, the original minimum numerical aperture (NA) requires for the latter OL represented by: $\mathrm{NA}_{\min} = \sin^{-1}(\frac{\theta_{FoV}}{2}) = 0.855$ (in the air); then $\mathrm{NA}_{\min} = 0.636$ in "X" pattern, which meets the requirements of OL with NA = 0.75.

4. Polarization state reconstruction from the vectorial meta-holography

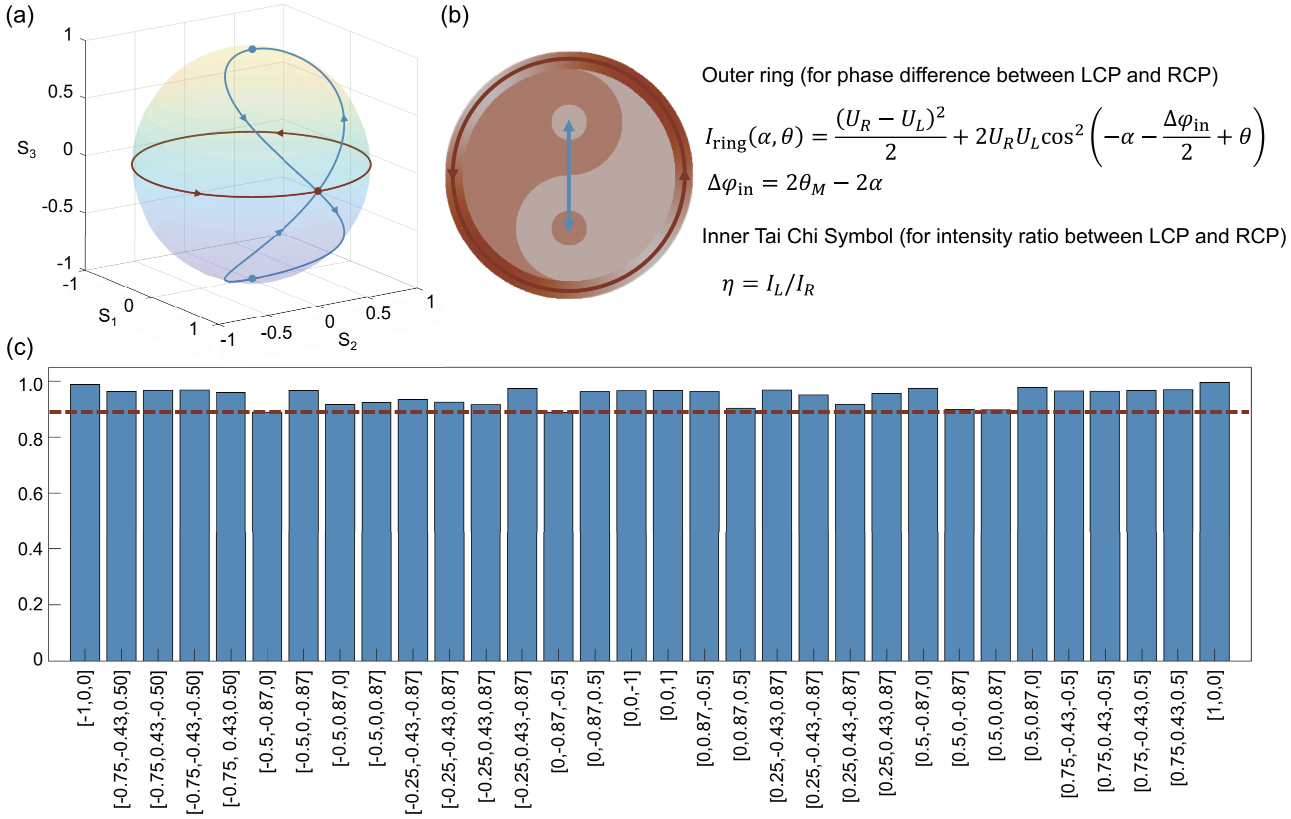

Fig. S4 The principle of polarization state reconstruction. a, Effect of half-wave plate (HWP) and quarter-wave plate (QWP) for horizontally polarized $|H\rangle$ state incidence: When $|H\rangle$ state is incident, the polarization state undergoes a trajectory on the Poincaré sphere after passing through the HWP and QWP. **b,** Method for extraction of polarization states from vectorial meta-holography. The ring is extracted to differentiate the phase difference between LCP and RCP, while the Tai Chi Symbol is analyzed for the intensity ratio between LCP and RCP, which can be converted into points on the Poincaré sphere. **c,** The fidelity of state reconstruction in up to 32 distinct polarization states on the Poincaré sphere. The coordinates are shown as $[s_1, s_2, s_3]$, where $s_0 = 1$.

The extracted amplitude ratio and phase difference from vectorial holographic image can reconstruct the encoded polarization state. Facing the direction of incidence, if define the orientation of the fast axis as the rotation angle. On the Poincaré sphere, the red dot in Fig. S4a is as the starting point (horizontal polarization $|H\rangle$). While the photon passing through a HWP with a rotation angle of β, the trajectory on the sphere is a closed circle running counterclockwise along the equator in red, with a angle of 4β. While passing through a QWP of β, the trajectory is a closed loop in blue, running along the "8" shape, with the absolute values of the slopes of both longitude and latitude changing by 2β. When β undergoes π, the polarization will be back to the origin under both of the manipulation. The north (south) pole corresponds to RCP (LCP).

To generate arbitrary $|\psi_s^+\rangle$, we rotate the HWP (QWP) in equal steps of $\beta = \frac{\pi}{12}$ (ranging $[0, \frac{\pi}{2}]$ ($[-\frac{\pi}{2}, \frac{\pi}{2}]$)) to measure the holographic images. This results in $7 \times 13 = 91$ data points. At each angle of HWP, the HWP is the same at 0 and π, and then each QWP forms a complete

"8" shape. The QWP only needs to be moved from $-\frac{\pi}{4}$ to $\frac{\pi}{4}$ to cover half of the "8" shape over the entire Poincaré plane. Each pole point is measured 5 times, so the independent data points are $91 - 13 - 6 \times 6 - 5 \times 2 = 32$.

As for sufficient precision of state reconstruction, we have mildly reduced range of the rings for analysis. In Fig. S4b, after background subtraction, we perform the angular integration of intensity for the ring. Subsequently, we apply one-dimensional Fourier filtering, followed by fitting with cosine functions to obtain the phase difference between incident LCP and RCP ($\Delta\varphi_{\text{in}} = 2\theta_M - 2\alpha$, where $\alpha \neq 0$, in this case, the measurement angle value can be shifted by α). For the Tai Chi Symbol, we collect the intensity values corresponding to LCP and RCP incidence separately. Utilizing an iterative method with activation function to reduce sensitivity to speckle noise, we obtain the square of amplitude ratio (intensity ratio) between RCP and LCP ($\eta^{-1} = I_R/I_L$).

Then, we calculate the angles of polarization ellipse using formulas: $\psi = \frac{1}{2}\Delta\varphi_{\text{in}}$, $\chi = \frac{1}{2}\arcsin\frac{\eta-1}{\eta+1}$. Subsequently, we transform the angles to Stokes parameters (s_0, s_1, s_2, s_3) on the Poincaré sphere. The (ψ, χ) can represent the Stokes parameters (s_0, s_1, s_2, s_3) as the coordinates on the sphere: $s_0 = I$, $s_1 = I\cos 2\psi\cos 2\chi$, $s_2 = I\sin 2\psi\cos 2\chi$, and $s_3 = I\sin 2\chi$, where $I = a_L^2 + a_R^2 = 1$. The five sets namely (1,0,0,-1), (1,0,0,1), (1,1,0,0), (1,$-\frac{1}{2}$,$\frac{\sqrt{3}}{2}$,0), (1,0,$\frac{\sqrt{3}}{2}$,$-\frac{1}{2}$) in Stokes parameters, are displayed in main text Fig. 3c. Through the analysis of input and extraction, we map the fidelity of polarization state reconstruction distributed across the entire polarization Poincaré sphere. The fidelity, representing the correlation between the incident and the reconstructed Stokes vectors $\mathbf{S}$ and $\mathbf{S}_r$ on the Poincaré sphere, exceeds 88.84% at the lowest (red dashed line) and 94.78%±3.04% on average. Thus, the color of the dots on the Poincaré sphere is close.

5. Determining the ϕ_q from the spontaneous parametric down-conversion (SPDC)

Focusing on the interface of two orthogonal β-barium borate (BBO) crystals, we identify the entangled photon-pairs generated by Type-I

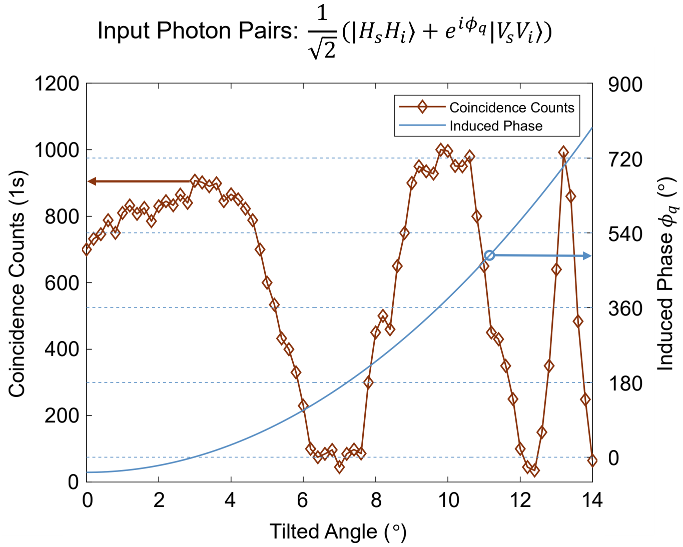

Fig. S5 ϕ_q **is related to the tilted angle of the zero-order QWP.** The red diamonds represent the coincidence counts per second measured under $|+_s+_i\rangle$ for each 0.2° steps in counterclockwise, while the blue line represents the induced phase delay ϕ_q.

SPDC, with the same amplitudes triggered in different states. Thus the wave function can be written as $|\Phi^+\rangle = \frac{1}{\sqrt{2}}(|H_sH_i\rangle + e^{i\phi_q}|V_sV_i\rangle)$. We select a new pair of orthogonal states $(|+\rangle, |-\rangle)$ both for measurements at the idler and signal terminals. These measurement states are located on the equator of the Poincaré sphere (LPs), and the transform relationship between them is:

$$|H\rangle = \cos\xi\,|-\rangle - \sin\xi\,|+\rangle\,, |V\rangle = \sin\xi\,|-\rangle + \cos\xi\,|+\rangle\,,$$

where ξ is the angle between the new state and the horizontal polarization state. Then the wave function becomes:

$$|\Phi^+\rangle = (\cos^2\xi + e^{i\phi_q}\sin^2\xi)\,|-_s-_i\rangle + (e^{i\phi_q} - 1)\cos\xi\sin\xi\,|+_s-_i\rangle + (e^{i\phi_q} - 1)\cos\xi\sin\xi\,|-_s+_i\rangle + (\sin^2\xi + e^{i\phi_q}\cos^2\xi)\,|+_s+_i\rangle\,.$$

The intensity when triggering to the same or different polarization states at both terminals respectively is: $I_{\text{same}} = \frac{1}{2}(\cos^4\xi + \sin^4\xi + \frac{1}{2}\sin^2 2\xi)$, $I_{\text{diff}} = \frac{1}{4}(1 - \cos\phi_q)\sin^2 2\xi$. When expressed as $\sin\zeta = \sin\frac{\phi_q}{2}\sin 2\alpha$, their ratio can be denoted as $\frac{I_{\text{diff}}}{I_{\text{same}}} = \tan\zeta$. When ϕ_q is 0°, $\tan\zeta = 0$, indicating complete extinction; and when ξ is ±45° (i.e., when the HWP is set to ±22.5°), $\tan\zeta = \pm\sin\frac{\phi_q}{2}$, implying that when ϕ_q is sufficiently large, the ratio between the two sets of measurement results can undergo arbitrary changes.

The quartz zero-order QWP manipulates the polarization based on the birefringence of quartz crystal for ordinary (o) and extraordinary (e) light. When the incident wavelength is 404 nm, their refractive indices are respectively: $n_o = 1.5572$, $n_e = 1.5668$. We directly connect the signal and idler photons to $SPCM_1$ and $SPCM_2$ via SMFs, single photon count module (SPCM). According to the coincidence counts in state $|+_s+_i\rangle$ with $\xi = 45°$, while rotating the extraordinary axis of QWP to be horizontal and tilting it around the vertical axis, we observe a continuous induced phase ϕ_{delay} resulted the different ϕ_q, as shown in the Fig. S5. At approximately 3.0°, 10.0°, and 13.0°, we achieve $\phi_q = 0$; while at around 7.0° and 12.4°, $\phi_q = \pi$ is obtained[S8]. This method is also used in Ref. [S9] for reconstructing the phase profile.

6. Comparison of imaging by using raster scanning system and electron-multiplying charge coupled device (EMCCD)

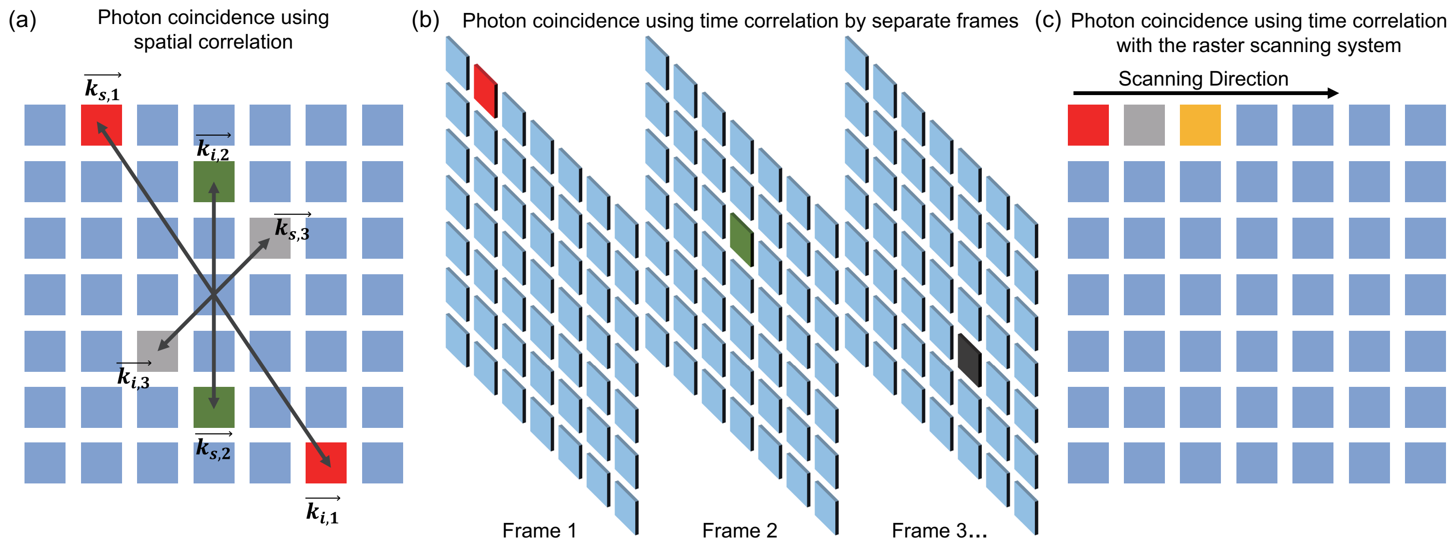

Fig. S6 Diagrams for three methods in quantum imaging. a, Photon coincidence using spatial correlation. **b,** Photon coincidence using time correlation. **c,** Photon coincidence using the MMF-based raster scanning system in this work.

When the photons is incident, the zero-order output occupies fewer pixels, resulting in significant intensity differences compared to individual pixels in the holographic image area. Captured by a charge coupled device (CCD), the zero-order output tends to saturate due to insufficient pixel depth, while the image is significantly affected by readout noise. Therefore, it is challenging to simultaneously measure the intensity values between the zero-order output and the holographic image.

In the single frame case, the EMCCD is well-suited for single photon meta-holography benefited from its high pixel density, high signal-to-noise ratio (SNR) and low readout electronic noise at the 808 nm wavelength approximately 75% high quantum efficiency[S10]. We set the parameters of the EMCCD to the electron-multiplying (EM) gain of 1000, the working temperature of -40°C, with a pixel pitch of 13 μm. At the focus of the zero-order spot, both the coupling efficiency and the clarity of holographic image are highest. In the experiment, we take the image size of 200×200 pixels on EMCCD with the dimensions of 2.6×2.6 mm^2. Through low-noise integration of dim light field incidence, we obtain photon counts for holographic images and zero-order output as 2.68×10^7 and 2.59×10^7 in 0.5 s, yielding the experimental diffraction efficiency of 50.9%.

Long-term operation of EMCCD results in heat accumulation, causing an increase in the temperature of the camera, amplifying electrical noise, and affecting the required counts for imaging due to exceeding a single electron threshold. Moreover, due to serial readout, over-saturation of a single pixel may propagate to an entire row of pixels, leading to the smearing effect. While using built-in water cooling can alleviate the issue, smearing effect and salt-and-pepper noise are still observable.

For correlation holography of entanglement-controlled vectorial meta-holography (ECVMH), the required nanosecond resolution is not achievable with EMCCD. The multi-mode fiber (MMF)-based raster scanning system (RSS) primarily comprises of two orthogonal fixed motorized linear translation stages, two SPCMs, and an MMF imaging fiber, providing a nanosecond-scale time resolution, which is notably superior to the former (millisecond-scale). It features an approximate minimum pixel pitch of 25 μm (equivalent to the radius of the MMF core) and boasts a higher quantum efficiency of 80% with lower readout noise. On the one hand, the increased pixel area reduces the FoV (see Supplementary Information 3) for environmental noise entering the detector's pixel units. On the other hand, the simultaneity of entangled photon-pairs allows for coincidence counting, which effectively filters out noise photons and enhances the SNR through quantum entanglement.

When using a classical light source, an image with sufficient intensity is captured requiring only a few snapshots using a camera. When using a quantum source, the image is obtained by measuring the coincidence counts of the photon pairs. Within a single frame, only one pair of photons can be detected. Each individual coincidence count must be collected within a very short time window to exclude the accidental coincidence with other uncorrelated photons. For instance, the images contain $m \times m$ pixels, and each pixels detect N coincidence counts. To quantify computational resources for the data processing and the storage, we compare two main methods for quantum imaging, as well as our multi-mode fiber (MMF)-based raster scanning system:

1. Photon coincidence using spatial correlation. For photon pairs generated by the spontaneous parametric down-conversion (SPDC), they are momentum-correlated due to the nonlinear wavevector relation $\mathbf{k}_s + \mathbf{k}_i = \mathbf{k}_p$, where s, i, and p represent the signal, idler, and pump photons, respectively[S11].Thus, they are symmetrically located on the imaging array of a camera, as shown in Fig. S6a. Using this method, multiple photon pairs can be collected simultaneously in a single frame, when the momentum distribution of photon pairs satisfies the sparsity condition on the imaging array. For the typical quantum imaging device like the electron-multiplying CCD, *the pump laser power and camera exposure time are set to ensure an optimum peak count probability of 0.2*[S12].That is to say, the effective average photon count on the imaging array is up to 0.2 counts/pixel/frame to satisfy the sparse distribution condition. The coincidence

measurements need to capture N frames ($N \times m \times m$ pixels), To calculate coincidence counts and to store all the frames, the required computational and storage resources are both $\sim Nm^2$. For $N = 1024, m = 1024$, they are 0.31 GB and 0.63 GB, respectively.

2. Photon coincidence using time correlation. The idler photon from SPDC is directed to a SPCM to trigger the camera like an intensified CCD (ICCD) to capture a single frame of the signal photon[S13]. To avoid receiving more than one photon pair, the pump laser power and the camera exposure time must be adjusted so that the optimal peak count probability is 1 count/frame, as shown in Fig. S6b. To achieve the same amount of coincidence counts as that using spatial correlation in method 1, the coincidence measurements need to capture $\sim Nm^2$ frames. Therefore, the needed computational resources are $\sim$ 1, but the required storage resources are $\sim Nm^4$. For $N = 1024, m = 1024$, the frames, the computational and storage resources are 1×10^9, 1 bit and 256 GB (after compression), respectively.

3. Photon coincidence using our method. The coincidence counts are raster scanned and measured by two SPCMs, as shown in Fig. S6c. To achieve the same amount of coincidence counts as that using spatial correlation in method 1, the required computational resource is $\sim$ 1 bit, while the required storage resources are $\sim Nm^2$. For $N = 1024, m = 1024$, the frames, the computational and storage resources are greatly reduced to 1, 1 bit and 0.25 GB, respectively.

Compared to the case of using a camera, the raster scanning system cannot capture photons of multiple pixels simultaneously. It will take a long time to capture the image if the number of pixels is very big. At the same time, the repetition resolution of a motorized linear translation stage cannot preserve high accuracy for a long time, which may cause unavoidable distortion in the images. The problem can be mitigated by replacing the slow translation stage with fast micro-electromechanical systems.

7. The entanglement correlation of SPDC source and the zero-order output

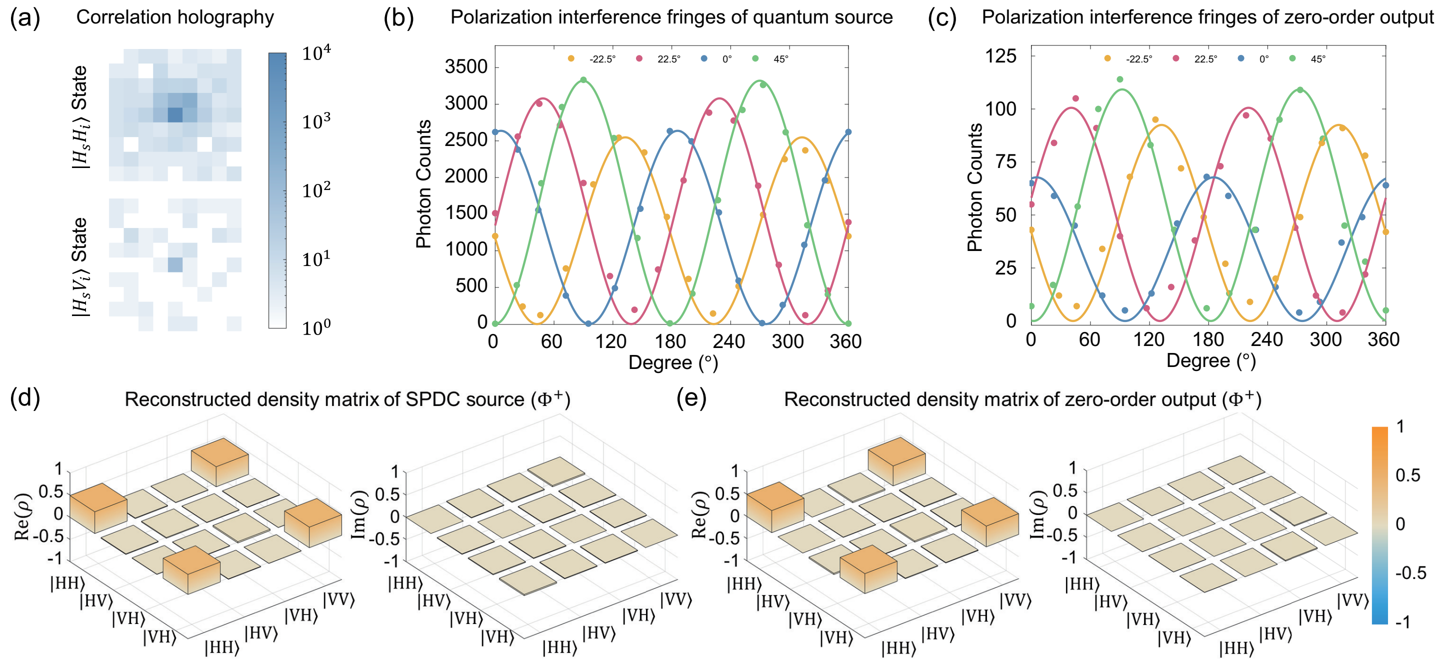

Fig. S7 The preservation of entanglement correlation after metasurface. a, Correlation of entangled photon-pairs under a set of measurement basis of zero-order output. **b,** and **c,** The two-photon polarization interference fringes for original SPDC source or with idler and zero-order photons, respectively. The joint coincidences (points) are fitted with cosine function (solid line) with the LP states of -45°, 45°, 0°, and 90°, and the signal LP state ranges from 0° to 360°. **d,** and **e,** Reconstructed density matrices for original SPDC source or with idler and zero-order photons, respectively.

First, we use the RSS to locate the position of the zero-order spot to certain the imaging plane. By collecting the zero-order output, the coincidence counts can be maximized through rotating the HWP and QWP. The resulting vectorial meta-holography is consistent the polarization of the incident states in the experiment. With future design, only a small portion of energy is used for ECVMH, leaving the remaining part available for quantum information applications. Thus we measure the properties of the entangled photon-pairs $|\Phi^+\rangle = \frac{1}{\sqrt{2}}(|H_s H_i\rangle + |V_s V_i\rangle)$. After scanning, we obtain the correlation holographic image with 10 nm interference filter (IF) as shown in Fig. S7a. After 20 s integration, the zero-order output coincidence for $|H_s H_i\rangle$ and $|H_s V_i\rangle$ are 8518 and 89, respectively (approximately 96:1 extinction ratio).

Next, we measure the two-photon coincidence counts of SPDC source and zero-order output to characterize the entangled state under different measurement states (3 nm IF), as shown in Fig. S7b and c, respectively. We characterize the quantum properties between the signal and idler photons using the Clauser-Horne-Shimony-Holt (CHSH) Bell-type inequality, which typically assesses the input polarization entanglement state. In experiment, the correlation coefficients $E(\alpha', \beta')$ values are obtained by measuring joint coincidences as:

$$E\left(\alpha', \beta'\right) = \frac{C(\alpha', \beta') + C(\alpha'_\perp, \beta'_\perp) - C(\alpha'_\perp, \beta') - C(\alpha', \beta'_\perp)}{C(\alpha', \beta') + C(\alpha'_\perp, \beta'_\perp) + C(\alpha'_\perp, \beta') + C(\alpha', \beta'_\perp)},$$

where $C(\alpha', \beta')$ is the measured coincidences for the projective measurement in the polarization states of $(\mathrm{HWP}_s(\alpha'/2), \mathrm{HWP}_i(\beta'/2))$, where α' ($\alpha\prime_\perp$) and β' ($\beta'_\perp$) are the polarization angles of HWP, $\alpha'_\perp = \alpha' + 90°$ and $\beta'_\perp = \beta' + 90°$. We chose $\alpha'_1 = -45°, \alpha'_2 = 0°, \beta'_1 = -22.5°, \beta'_2 = 22.5°$. We perform a Bell test using CHSH inequality ($S \leq 2$) with original SPDC source, and obtain the CHSH value of $S = 2.703 \pm 0.019$ (violates the local hidden variable bound by $37.0\sigma_{\text{std}}$), which for idler and zero-order photons is $S = 2.644 \pm 0.058$ ($11.1\sigma_{\text{std}}$).

With the same configuration as described above, the angle α' of HWP_1 is set to -22.5°, 22.5°, 0° and 45°. Then, the angle β' of HWP_2 is rotated to obtain the coincidence measurement $C(\alpha', \beta')$ at each point in 5 s. It can be observed that the zero-order output generated by the signal photon passing through the metasurface still preserves the entanglement. We collect the photon from the zero-order output using the MMF without the polarizer, and correlating them with the idler photon, whose trend is consistent with the pattern observed in Fig. S7b. We measure the correspond interference visibility of 0.886 ± 0.007, 0.916 ± 0.006, 0.986 ± 0.003, 0.994 ± 0.002 (for SPDC source), and 0.820 ± 0.043, 0.892 ± 0.036, 0.889 ± 0.046, 0.912 ± 0.033 (for zero-order output).

We reconstruct the density matrix of quantum source and zero-order output with the maximum likelihood estimation method based on 36 two-photon coincidence counts. The fidelity is defined as $F(\rho, \widetilde{\rho}) = [\mathrm{Tr}(\sqrt{\sqrt{\widetilde{\rho}}\rho\sqrt{\widetilde{\rho}}})]^2$, where $\widetilde{\rho}$ is the reconstructed density matrix as shown in Fig. S7d and e, and ρ is the density matrix of the expected entangled state. The fidelities between the reconstructed and expected entangled state of quantum source and zero-order output are 0.933 ± 0.002 and 0.929 ± 0.006, respectively. Therefore, owing to the sensitivity enhancement and improved imaging quality of quantum holography, fewer photons are required for the extraction, while the excess photons passing through the zero-order output, remain a quantum entangled source.

8. The imaging SNR of single photon holography and correlation holography

Table S1 The experimental (*italic*) and estimated imaging SNR from the coincidence counts and background. The errors for $\eta_s\eta_i\overline{I_i^{\text{coin}}}$, $C_{\text{accidental}}$, and $\overline{I_s^b}$ are estimated using the Poisson distribution. The SNRs in the last three columns are calculated based on the estimation, and the SNRs in *italic* are calculated from the experimental holographic images.

Counts in 600s	$\eta_s\eta_i\overline{I_i^{\text{coin}}}$	$C_{\text{accidental}}$	$\overline{I_s^b}$	SNR_{coin} (dB)	SNR_{sin} (dB)	ΔSNR (dB)
LCP	352.50±18.77	29.43±5.42	6371.70±79.82	11.13±0.77(*10.78*)	1.06±0.03(*0.72*)	10.07±0.75(*10.06*)
RCP	315.30±17.75	38.93±6.24	6387.52±79.92	9.59±0.65(*9.08*)	0.95±0.02(*0.01*)	8.64±0.61(*9.07*)

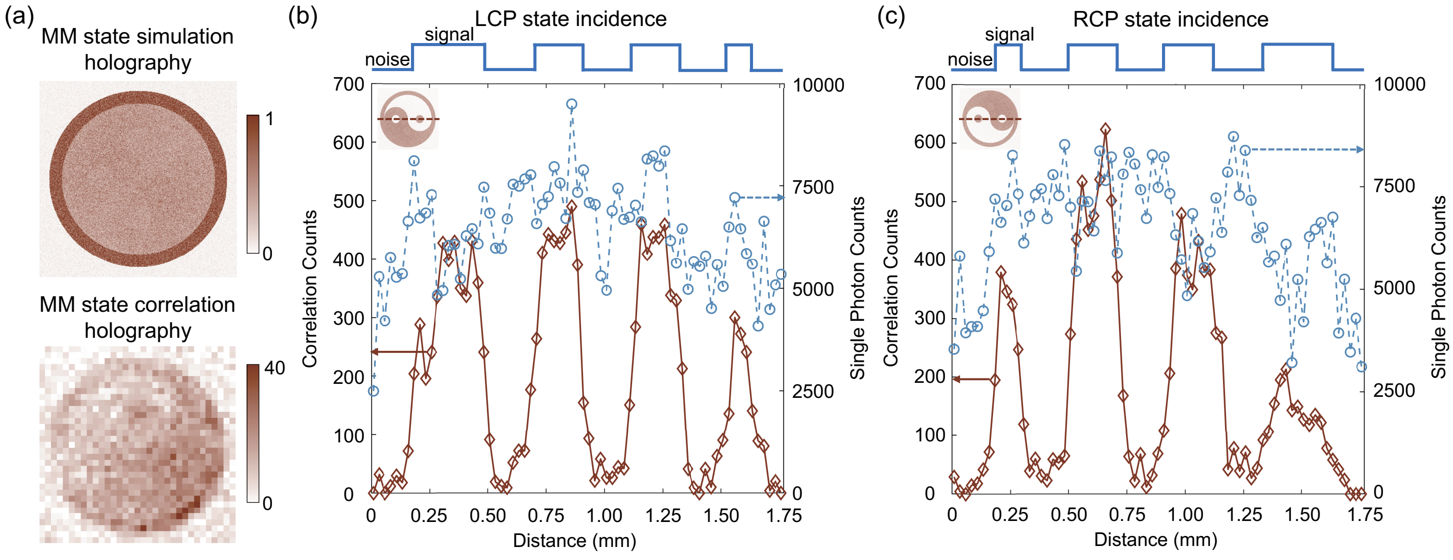

Fig. S8 The ECVMH of the MM state and the SNR enhancement. a, The ECVMH result with the MM state incidence. The ECVMH is acquired with 40 s integration time, 33×33 pixels, and 50 μm scanning step. **b,** and **c,** The SNR enhancement of ECVMH along the central line. The results include the single photon (blue circles) and the correlation holography (red diamonds), respectively. Triggering the idler photon to $|H_i\rangle$ or $|V_i\rangle$ state, the signal photon is in $|L_s\rangle$ or $|R_s\rangle$ state with 600 s integration time, 1×71 pixels, and 25 μm scanning steps. The bright area (marked as "*signal*") and dark area (marked as "*noise*") of Tai Chi Symbol are shown in blue lines on the upper for the incidence with LCP and RCP states, respectively.

Given the significant difference in the counts of idler and signal photons, the primary source of noise arises from the random coincidences between idler photons and noise photons at the signal terminal. Entangled photon-pairs are generated through two orthogonal oriented BBO crystals, with the probabilities of SPDC processes in two crystals being p_{I} and p_{II} respectively. $\text{SPCM}_{1(2)}$ is located at the signal (idler) terminal, and the qauntum efficiencies of paths are $\eta_{s(i)}$. Therefore, the single photon counts are $D_{s(i)} = (p_{\text{I}} + p_{\text{II}})\eta_{s(i)}$, and the coincidence counts are $D = (p_{\text{I}} + p_{\text{II}})\eta_s\eta_i$.

While measuring the background, the counting probability of SPCM_1 is p_b, and the single photon counts are $D_{b,s} = p_b\eta_s$. The coincidence counts D_b are obtained when the idler photons are in the maximally mixed state, and $D_b = (\sqrt{p_b p_{\text{I}}} + \sqrt{p_b p_{\text{II}}})\eta_s\eta_i$. While triggering the idler photon to different polarization states (*i.e.* horizontally or vertically polarized state), $D_{b,s}$ remains unchanged, while $D_{b,H(V)} = \sqrt{p_b p_{\text{I(II)}}}\eta_s\eta_i$. Typically, we ensure $p_{\text{I}} \approx p_{\text{II}}$ to achieve the desired entangled state, thus $D_{b,H(V)} \approx \frac{1}{2}D_b$. D_b is the coincidence counts between the maximally mixed state in the idler terminal and background in the signal terminal, and $D_{b,H(V)}$ is the coincidence counts between the pure state in the idler terminal and background in the signal terminal. In contrast, the single photon counts of background in the signal terminal is equal to $D_{b,s}$ no matter with the incidence for the MM state or the pure state in the idler terminal.

We assume that the total intensities of the signal and idler photons are given by $I_s = \eta_s I_s^{\text{coin}} + I_s^b$ and $I^i = \eta_i I_i^{\text{coin}} + I_i^b$, where the total intensities are related to the coincidence intensity of the bi-photons $I_{s(i)}^{\text{coin}}$ and the background intensity $I_{s(i)}^b$. The covariance between the intensities is $\text{cov}(I_s, I_i) = \frac{1}{N-1}\sum_k^N (I_s^k - \overline{I_s^k})(I_i^k - \overline{I_i^k})$, where N is the number of frames (which can be considered as N time bins), the superscript k is frame index, and $\overline{I}$ is the average intensity of I in such time bin. Given that the number of imaging pixels (33 × 33) is much greater than one, the signal photon intensity I_s of each pixel is much lower than I_i, and $I_i \gg I_{s(i)}^b$. We now expand and simplify the covariance as follows:

$$\begin{aligned}
\text{cov}(I_s, I_i) &= \frac{1}{N-1}\sum_N^k (I_s^k - \overline{I_s^k})(I_i^k - \overline{I_i^k}) \approx \overline{(I_s - \overline{I_s})(I_i - \overline{I_i})} = \overline{I_s I_i} - \overline{I_s}\cdot\overline{I_i} \\
&= \overline{(\eta_s I_s^{\text{coin}} + I_s^b)(\eta_i I_i^{\text{coin}} + I_i^b)} - \overline{(\eta_s I_s^{\text{coin}} + I_s^b)}\cdot\overline{(\eta_i I_i^{\text{coin}} + I_i^b)} \\
&= \overline{(\eta_s I_s^{\text{coin}} + I_s^b)(\eta_i I_i^{\text{coin}} + I_i^b)} - ((\eta_s \overline{I_s^{\text{coin}}} + \overline{I_s^b})\cdot(\eta_i \overline{I_i^{\text{coin}}} + \overline{I_i^b})) \\
&\approx \eta_s\eta_i \overline{I_s^{\text{coin}} I_i^{\text{coin}}} + \eta_i \overline{I_i^{\text{coin}} I_s^b} + \overline{I_s^b I_i^b} - \eta_s\eta_i \overline{I_s^{\text{coin}}}\cdot\overline{I_i^{\text{coin}}} - \eta_i \overline{I_i^{\text{coin}}}\cdot\overline{I_s^b} - \overline{I_i^b}\cdot\overline{I_s^b}.
\end{aligned}$$

Since the background noise is uncorrelated between these relatively distant regions, and both the coincidence intensity and the background noise follow a Poisson distribution (where the variance equals the mean), we obtain:

$$\begin{aligned}
\text{cov}(I_s, I_i) &\approx \eta_s\eta_i \overline{I_s^{\text{coin}} I_i^{\text{coin}}} - \eta_s\eta_i \overline{I_s^{\text{coin}}}\cdot\overline{I_i^{\text{coin}}} + \eta_i \overline{I_i^{\text{coin}} I_s^b} - \eta_i \overline{I_i^{\text{coin}}}\cdot\overline{I_s^b} \\
&= \eta_s\eta_i \text{cov}(I_s^{\text{coin}}, I_i^{\text{coin}}) + \eta_i \text{cov}(I_i^{\text{coin}}, I_s^b) \\
&\leq \eta_s\eta_i \overline{I^{\text{coin}}} + \eta_i\sqrt{\overline{I^{\text{coin}}}\cdot\overline{I_s^b}}, \text{ while the equality holds when } I^{\text{coin}} \text{ and } I_s^b \text{are identically distributed, evidently it is hard.}
\end{aligned}$$

Thus, the lower-bound of the SNR of coincidence counts is:

$$\text{SNR}_{\text{coin}} = 10\log\frac{I_{\text{signal}}}{I_{\text{background}}} = 10\log\frac{\eta_s\eta_i\text{cov}(I_s^{\text{coin}}, I_i^{\text{coin}}) + \eta_i\text{cov}(I_i^{\text{coin}}, I_s^b)}{\eta_i\text{cov}(I_i^{\text{coin}}, I_s^b)} \geq 10\log(\eta_s\sqrt{\frac{\overline{I^{\text{coin}}}}{\overline{I_s^b}}} + 1),$$

In the experiment, the covariance between I_i^{coin} and I_s^b can be assume to be a constant $C_{\text{accidental}}$, termed as accidental coincidence, which is smaller than either of them. Thus, $\text{SNR}_{\text{coin}} \approx 10\log(\frac{\eta_s\eta_i\overline{I^{\text{coin}}}}{C_{\text{accidental}}} + 1)$. The SNR of the image for a single signal photon is:

$$\text{SNR}_{\text{sin}} = 10\log\frac{I_{\text{signal}}}{I_{\text{background}}} = 10\log(\frac{\eta_s\overline{I^{\text{coin}}}}{\overline{I_s^b}} + 1).$$

To estimate the SNR enhancement, we use data from Fig. S8b (600s integration), shown in Table S1. Calculating the SNR for the image of a single signal photon is challenging, because the distributed intensity $\eta_s\overline{I^{\text{coin}}}$ is much smaller than $\overline{I_s^b}$ in each pixel. Based on empirical estimation, the single-photon counting is ~ 5 times greater than the coincidence counts in our setup, e.g. $\eta_i \approx 0.2$. The SNR enhancement is $\Delta\text{SNR} = \text{SNR}_{\text{coin}} - \text{SNR}_{\text{sin}}$, highlighted in the last column. The relatively high error arises from accidental coincidences, which can be mitigated by increasing the integration time. This suggests that the high SNR enhancement is due to the quantum correlation effectively preserving the signal despite the background noise. The highest SNR enhancement occurs under the conditions of $\eta_s\overline{I_i^{\text{coin}}} \gg \overline{I_s^b}$, $\eta_s\eta_i\overline{I_i^{\text{coin}}} \gg C_{\text{accidental}}$ and $\eta_i \approx 1$, we get the upper limit of $\Delta\text{SNR} = 10\log\overline{I_s^b}/C_{\text{accidental}} \approx 23.35$dB (for LCP state).

The intensities distribution of holographic images after the polarizer is complementary, when the orthogonal polarization states is incident. When the signal photon collapses to the maximally mixed (MM) state, the resulting correlation holography matches the theoretical simulations, as shown in Fig. S8a. Quantum correlation can significantly enhance the SNR. We perform with photon-pairs $|\Phi_{LR}^+\rangle = 1/\sqrt{2}(|L_s H_i\rangle + |R_s V_i\rangle)$ to demonstrate ECVMH. From Fig. S8b and c, we scan a line across the center of the Tai Chi Symbol of the LCP (RCP) based ECVMH, which needs almost 12 hours.

The SNR is calculated by $\text{SNR} = 10\log(I_{\text{singal}}/I_{\text{backgroud}})$, where I_{singal} and $I_{\text{backgroud}}$ are the average counts in the "*signal*" and "*noise*" components. The blue lines on the upper of the Fig. S8b and S8c show the theoretical counts along the line of Tai Chi Symbol region (upper left inset) within the same time window. **The average counts in these components contain background noise, which leads to the difference in the calculation between the experiment and estimation.** It is evident that the SNR of the correlation holography is significantly enhanced (almost 10 dB). For comparison, with the LCP state incidence, the SNR obtained from simulation is 11.69 dB, with 0.72 dB for the single photon holography and 10.78 dB for the correlation holography, and the SNRs are 10.87 dB, 0.01 dB, and 9.08 dB with the RCP state incidence, respectively. The experimental enhancement is lower than estimated because extracting the signal from single-photon holography is challenging.

Now, moving on to the RSS, this scanning concept is already utilized in Light Detection and Ranging (LiDAR) technology[S14]. The core of the MMF (50/125) has a corresponding numerical aperture (NA) of 0.20, while the EMCCD with 13 μm pixels has an NA of 0.59, calculated

based on the C-mount and a focus depth of 0.69 inches, resulting in a higher I_b^s. In comparison to the RSS, the salt-and-pepper noise and electrical noise from the camera also increase I_b^s, thereby reducing the SNR[S15]. The integration time required by the RSS to achieve the same level of SNR is 1-2 orders of magnitude shorter than other remarkable works in quantum imaging using different types of quantum cameras, including ICCD[S16], EMCCD[S9], timepix3 ASIC cameras (TPX3CAMs)[S17], single-photon avalanche detectors (SPAD) camera[S18] and qCMOS camera[S15]. With improvements in the brightness of the quantum source, the RSS for its simplicity, economy and flexibility could become even more advantageous for quantum imaging.

9. The conversion between two quantum entanglement states

To demonstrate the polarization measurement results under different entangled states after polarization conversion, we continue to use the amplitude ratio and phase difference extracted from the holographic images for analysis. We generate the entangle state is $|\Phi_{LR}^{+}\rangle = 1/\sqrt{2}(|L_s H_i\rangle + |R_s V_i\rangle)$ in experiment, converting it to the eigenstates $|\psi_s^{\pm}\rangle$ (the signal photon changes) and $|\phi_i^{\pm}\rangle$ (the idler photon remains) of new observable[S19], such that:

$$\begin{pmatrix}\psi_s^+\\ \psi_s^-\end{pmatrix} = A\begin{pmatrix}L_s\\ R_s\end{pmatrix} = \begin{pmatrix}a & b\\ c & d\end{pmatrix}\begin{pmatrix}L_s\\ R_s\end{pmatrix}, \begin{pmatrix}\phi_i^+\\ \phi_i^-\end{pmatrix} = \begin{pmatrix}H_i\\ V_i\end{pmatrix},$$

where A is unitary ($A^*A = I$, "$*$" is conjugate transpose and I is identity), with the definition $|\psi_s^{\pm}\rangle$ of in main text, A can be written as:

$$A = \begin{pmatrix}a & b\\ c & d\end{pmatrix} = \begin{pmatrix}a_L e^{i\Delta\varphi_{\text{in}}} & a_R\\ -a_R & a_L e^{-i\Delta\varphi_{\text{in}}}\end{pmatrix},$$

where a_L,a_R, and $\Delta\varphi_{\text{in}}$ denote the amplitudes of LCP, RCP components and their phase difference, respectively. Neglecting decoherence of polarization state and inherent losses from metasurface thus to $a_L^2 + a_R^2 = 1$, the entangle state can be written as (ignore the $1/\sqrt{2}$):

$$\begin{aligned}|\Phi_{LR}^{+}\rangle &= (a_L^2 e^{-2i\Delta\varphi_{\text{in}}} + a_R^2)\,|\psi_s^+\phi_i^+\rangle + a_L a_R(e^{i\Delta\varphi_{\text{in}}} - e^{-i\Delta\varphi_{\text{in}}})\,|\psi_s^-\phi_i^+\rangle + a_L a_R(e^{i\Delta\varphi_{\text{in}}} - e^{-i\Delta\varphi_{\text{in}}})\,|\psi_s^+\phi_i^-\rangle + (a_L^2 e^{2i\Delta\varphi_{\text{in}}} + a_R^2)\,|\psi_s^-\phi_i^-\rangle\\ &= (1 - 2ia_L^2 e^{-i\Delta\varphi_{\text{in}}}\sin\Delta\varphi_{\text{in}})\,|\psi_s^+\phi_i^+\rangle + 2ia_L a_R\sin\Delta\varphi_{\text{in}}\,|\psi_s^-\phi_i^+\rangle + 2ia_L a_R\sin\Delta\varphi_{\text{in}}\,|\psi_s^+\phi_i^-\rangle + (1 + 2ia_L^2 e^{i\Delta\varphi_{\text{in}}}\sin\Delta\varphi_{\text{in}})\,|\psi_s^-\phi_i^-\rangle\\ &= |\psi_s^+\phi_i^+\rangle + |\psi_s^-\phi_i^-\rangle + 2i\sin\Delta\varphi_{\text{in}}(-a_L^2 e^{-i\Delta\varphi_{\text{in}}}\,|\psi_s^+\phi_i^+\rangle + a_L a_R\,|\psi_s^-\phi_i^+\rangle + a_L a_R\,|\psi_s^+\phi_i^-\rangle + a_L^2 e^{i\Delta\varphi_{\text{in}}}\,|\psi_s^-\phi_i^-\rangle) \equiv |\Phi_\psi\rangle\,.\end{aligned}$$

While $\Delta\varphi_{\text{in}} = 0$,$|\Phi_\psi\rangle = 1/\sqrt{2}(|\psi_s^+\phi_i^+\rangle + |\psi_s^-\phi_i^-\rangle) \equiv |\Phi_\psi^+\rangle$; while $\Delta\varphi_{\text{in}} = \pm\pi/2$ and $a_L^2 = a_R^2 = 1/2$, $|\Phi_\psi\rangle = \pm i/\sqrt{2}(|\psi_s^-\phi_i^+\rangle + |\psi_s^+\phi_i^-\rangle) \equiv |\Psi_\psi^+\rangle$; otherwise, we get $|\Phi_\psi\rangle$ as the combination of those four eigenstates ($|\psi_s^{\pm}\phi_i^{\pm}\rangle$).

References

S1. Illingworth, J. & Kittler, J. A survey of the Hough transform. *Computer vision, graphics, and image processing* **44,** 87–116 (1988).

S2. Hsueh, C.-K. & Sawchuk, A. A. Computer-generated double-phase holograms. *Applied optics* **17,** 3874–3883 (1978).

S3. Huang, Z. & Cao, L. Quantitative phase imaging based on holography: trends and new perspectives. *Light: Science & Applications* **13,** 145 (2024).

S4. Li, Z., Wan, C., Dai, C. & Li, Z. Immersion-Triggered Active Switch for Spin-Decoupled Meta-Optics Multi-Display. *Small* **18,** 2205041 (2022).

S5. Brady, D. J., Choi, K., Marks, D. L., Horisaki, R. & Lim, S. Compressive holography. *Optics express* **17,** 13040–13049 (2009).

S6. Wu, J. *et al.* Single-shot lensless imaging with fresnel zone aperture and incoherent illumination. *Light: Science & Applications* **9,** 53 (2020).

S7. Li, X. *et al.* Athermally photoreduced graphene oxides for three-dimensional holographic images. *Nature Communications* **6,** 6984 (2015).

S8. Kwiat, P. G., Waks, E., White, A. G., Appelbaum, I. & Eberhard, P. H. Ultrabright source of polarization-entangled photons. *Physical Review A* **60,** R773 (1999).

S9. Defienne, H., Ndagano, B., Lyons, A. & Faccio, D. Polarization entanglement-enabled quantum holography. *Nature Physics* **17,** 591–597 (2021).

S10. Moreau, P.-A., Toninelli, E., Gregory, T. & Padgett, M. J. Imaging with quantum states of light. *Nature Reviews Physics* **1,** 367–380 (2019).

S11. Howell, J. C., Bennink, R. S., Bentley, S. J. & Boyd, R. W. Realization of the Einstein-Podolsky-Rosen Paradox Using Momentum- and Position-Entangled Photons from Spontaneous Parametric Down Conversion. *Physical review letters* **92,** 210403 (2004).

S12. Reichert, M., Defienne, H. & Fleischer, J. W. Massively parallel coincidence counting of high-dimensional entangled states. *Scientific reports* **8,** 7925 (2018).

S13. Altuzarra, C. *et al.* Imaging of polarization-sensitive metasurfaces with quantum entanglement. *Physical Review A* **99,** 020101 (2019).

S14. Dong, P. & Chen, Q. *LiDAR remote sensing and applications* (CRC Press, 2017).

S15. Roberts, K., Wolley, O., Gregory, T. & Padgett, M. A comparison between the measurement of quantum spatial correlations using qCMOS photon-number resolving and electron multiplying CCD camera technologies. *Scientific Reports* **14,** 14687 (2024).

S16. Zhou, J. *et al.* Metasurface enabled quantum edge detection. *Science advances* **6,** eabc4385 (2020).

S17. Thekkadath, G. *et al.* Intensity interferometry for holography with quantum and classical light. *Science Advances* **9,** eadh1439 (2023).

S18. Fan, Y. *et al.* Dual-channel quantum meta-hologram for display. *Advanced Photonics Nexus* **3,** 016011–016011 (2024).

S19. Nielsen, M. A. & Chuang, I. L. *Quantum computation and quantum information* (Cambridge university press, 2010).
